# Regulation of Drought and Salt Tolerance by OsSKL2 and OsASR1 in Rice

**DOI:** 10.1186/s12284-022-00592-2

**Published:** 2022-08-29

**Authors:** Yingli Jiang, Xiaojian Peng, Qin Zhang, Yuqing Liu, Aiqi Li, Beijiu Cheng, Jiandong Wu

**Affiliations:** National Engineering Laboratory of Crop Stress Resistance Breeding, School of Life Sciences, Anhui Agricultural University, Hefei, 230036 Anhui China

**Keywords:** Salt and drought tolerance, *Oryza sativa*, OsSKL2, Reactive oxygen species, Protein interactions, OsASR1

## Abstract

Abiotic stresses such as salinity and drought greatly impact the growth and production of crops worldwide. Here, a shikimate kinase-like 2 (*SKL2*) gene was cloned from rice and characterized for its regulatory function in salinity and drought tolerance. OsSKL2 was localized in the chloroplast, and its transcripts were significantly induced by drought and salinity stress as well as H_2_O_2_ and abscisic acid (ABA) treatment. Meanwhile, overexpression of *OsSKL2* in rice increased tolerance to salinity, drought and oxidative stress by increasing antioxidant enzyme activity, and reducing levels of H_2_O_2_, malondialdehyde, and relative electrolyte leakage. In contrast, RNAi-induced suppression of *OsSKL2* increased sensitivity to stress treatment. Interestingly, overexpression of *OsSKL2* also increased sensitivity to exogenous ABA, with an increase in reactive oxygen species (ROS) accumulation. Moreover, OsSKL2 was found to physically interact with OsASR1, a well-known chaperone-like protein, which also exhibited positive roles in salt and drought tolerance. A reduction in ROS production was also observed in leaves of *Nicotiana benthamiana* showing transient co-expression of OsSKL2 with OsASR1. Taken together, these findings suggest that OsSKL2 together with OsASR1 act as important regulatory factors that confer salt and drought tolerance in rice via ROS scavenging.

**Supplementary Information:**

The online version contains supplementary material available at 10.1186/s12284-022-00592-2.

## Background

Adverse environmental conditions, such as high salinity and drought, are major abiotic stresses affecting plant growth and agricultural productively. In response, plants have evolved a number of physiological, morphological, cellular and molecular mechanisms (Zhu 2016). For example, reactive oxygen species (ROS), such as superoxide anion radicals (O_2_^−^), hydroxyl radicals (OH) and hydrogen peroxide (H_2_O_2_), are well known important signal molecules in a number of biological processes (Mittler et al. 2011), including responses to salinity and drought. However, excessive accumulation of ROS is toxic, causing oxidative damage to proteins, DNA and membrane lipids (Apel and Hirt 2004). To mitigate this effect and maintain cellular redox homeostasis, plants have therefore evolved efficient nonenzymatic detoxification methods involving, for example, ascorbic acid and flavonoids, as well as enzymatic mechanisms such as superoxide dismutase (SOD), catalase (CAT), glutathione peroxidase (GPX) and peroxidases (POD) activities, all of which aim to scavenge ROS (Mittler et al. 2011; Lee et al. 2016).

Considerable research has also shown the important roles of the shikimate pathway and aromatic amino acids in plant defenses and protection as well as plant cell structure, plant signaling and reproduction, and plant development (Pagnussat et al. 2005; Bonawitz and Chapple 2010; Achary et al. 2020). The shikimate pathway connects primary and secondary metabolism, beginning with the catalyzation of phosphoenolpyruvate (PEP) and erythrose-4-phosphate (E4P) via several enzymes including 3-deoxy-D-arabino-heptulosonate 7-phosphate synthase (DAHPS), 3-dehydroquinate synthase (DHQS), bifunctional enzyme 3-dehydroquinate dehydratase/shikimate dehydrogenase (DHQD/SD), Shikimate kinase (SK), 5-enolypyruvylshikimate 3-phosphate synthase (ESPS) and chorismate synthase (CS), and ending with the production of chorismate (Tohge et al. 2013). Chorismate is then transformed into the aromatic amino acids phenylalanine, tyrosine, and tryptophan, which serve as precursors for many vital compounds, such as lignin, flavonoids and anthocyanins. The shikimate pathway is also known to be activated under abiotic stress conditions, such as drought and salinity, resulting in the accumulation of high levels of aromatic amino acids and related secondary metabolites (Maeda and Dudareva 2012; Francini et al. 2019). However, despite these findings, the gene(s) encoding the catalyzing enzymes within the shikimate pathway, thereby contributing directly to stress tolerance in plants, has yet to be determined.

SK catalyzes the fifth enzymatic step of the shikimate pathway, with the phosphorylation of shikimate to shikimate-3-phosphate. It has been suggested that plant SKs are crucial in facilitating metabolic fluxes within the shikimate pathway towards the production of a broad range of secondary metabolites involved in plant growth, development, and stress responses (Herrmann 1995). Various gene numbers of SK isoforms have been identified in several species, and while *Escherichia coli* has only two SKs, most plant genomes contain multiple forms (Tohge et al. 2013). The *Arabidopsis* genome contains two genes (*AtSK1* and *AtSK2*) encoding functional SK enzymes, and two SK homologs, shikimate kinase-like 1 (*AtSKL1*) and 2 (*AtSKL2*), which have evolved from SK gene duplicates and do not possess SK enzyme activity in vitro (Fucile et al. 2008). Research has shown that *AtSKL1* is crucial for chloroplast development, while AtSKL2 has acquired a protein–protein interaction domain that is important for adaptive molecular evolution (Fucile et al. 2008; Xu et al. 2018). Meanwhile, three SK genes (*OsSK1*, *OsSK2* and *OsSK3*) and two SK homologs (*OsSKL1* and *OsSKL2*) have so far been identified in rice, and while these rice SK genes are thought to contribute to defense responses and panicle development (Kasai et al. 2005; Tohge et al. 2013), little is known about the function of *OsSKL1* and *OsSKL2.*

Abscisic acid-stress-ripening (ASR) proteins are plant-specific, small hydrophilic, low molecular weight proteins induced by ABA and various abiotic stresses, such as drought and salinity (González and Iusem 2014). Overexpression or ectopic expression of *ASRs* in rice, maize, foxtail millet and wheat was found to improve crop adaption to adverse external environments by regulating ROS homeostasis (Virlouvet et al. 2011; Li et al. 2016, 2017; Park et al. 2019; Qiu et al. 2021). Moreover, studies have also shown that *ASR* genes encode transcription factors that directly bind to the promoter of targets genes, improving stress tolerance during stress responses (Arenhart et al. 2014; Li et al. 2018). Interestingly, it has also been reported that ASR proteins possess chaperone-like activity that protects proteins from inactivation (Konrad and Bar-Zvi 2008; Li et al. 2017).

In this study, we characterized the function of *OsSKL2* as a salt and drought responsive gene, and demonstrated its important role in stress tolerance using *OsSKL2* overexpressing and knockdown plants. *OsSKL2* was found to positively regulate salt and drought tolerance via ROS homeostasis and ABA sensitivity through interactions with OsASR1, which plays a similar role in stress responses in rice. These results suggest a regulatory mechanism involving OsSKL2 and OsASR1, providing new insight into increased tolerance to salt and drought stress in plants.

## Results

### OsSKL2 is a Chloroplast-Localized Protein Induced by Several Abiotic Stresses

**Fig. 1:**
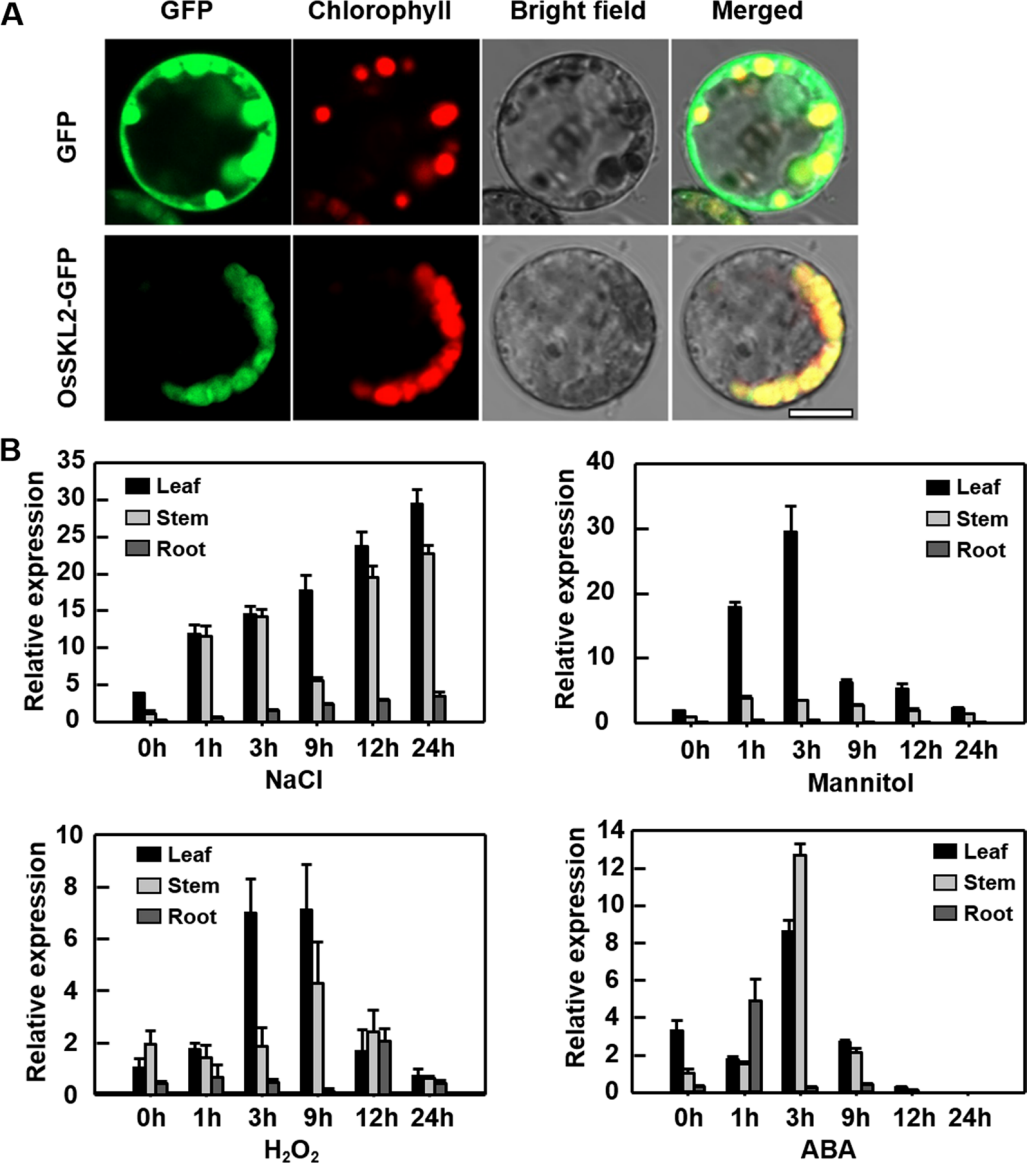
OsSKL2 subcellular localization and expression profiles. **A** Subcellular localization of OsSKL2 using a rice protoplast transient transformation system, showing chlorophyll localization (bar = 10 μm). **B** Inducible expression profiles of *OsSKL2* in response to various abiotic stresses. Two-week-old rice seedlings (Zhonghua11) were treated with 100 mM NaCl, 100 mM mannitol, 5 mM H_2_O_2,_ or 100 µM ABA. Root, stem and leaf samples were then harvested at 0, 1, 3, 9, 12, and 24 h after treatment, respectively. Expression levels of OsSKL2 were then determined with qRT-PCR using rice *Actin1* as an internal control

In previous transcriptome data analysis of rice under high salinity treatment, we identified *OsSKL2* and showed its high similarity to *AtSKL2* (Additional file 1: Fig. S1). Since plant SK and SK-like homologs (SKLs) have never been reported in response to abiotic stress, this prompted us to investigate the function of *OsSKL2* under stress conditions. First, the subcellular localization of OsSKL2 was analyzed by transforming rice leaf protoplasts with GFP-fused OsSKL2. In the control GFP protoplast, fluorescence was detected both in the cytoplasm and nucleus, while the OsSKL2-GFP fusion signal was observed only in the chloroplast (Fig. 1A), suggesting chloroplast localization. To further reveal the function of *OsSKL2*, qRT-PCR was performed to determine the transcriptional level of *OsSKL2* in rice leaf, stem and root samples following high salinity, mannitol, H_2_O_2_ and ABA treatments (Fig. 1B). No significant difference of *OsSKL2* expression was detected under normal condition (Additional file 1: Fig. S2). However, transcription levels were rapidly induced in the leaves following 1–3 h treatment with NaCl, PEG, H_2_O_2_ and ABA. In addition, varying transcriptional levels were detected in the stems and roots, with notably high levels following NaCl and ABA treatment. These results suggest that *OsSKL2* is responsive to multiple abiotic stresses.

### OsSKL2 Positively Regulates Salt and Drought Tolerance in Rice

To explore the role of *OsSKL2* in rice, we generated *OsSKL2* overexpressing (OE) and RNAi transgenic (ZH11 background) lines. Two independent OsSKL2-OE (OE3 and OE6) and OsSKL2-RNAi lines (Ri6 and Ri9) possessing different transcription levels were confirmed by qRT-PCR and selected for the following research (Additional file 1: Fig. S3). Since *OsSKL2* is a SK homolog (Fucile et al. 2008), we first chose to examine shikimate levels in the transgenic plants. Accordingly, compared with the wild-type (WT) plants, no obvious differences in the shikimate content were detected (Additional file 1: Fig. S4), suggesting that OsSKL2 does not possess encoded SK enzyme activity.

To confirm the function of *OsSKL2* in salt and drought resistance in rice, we therefore examined the performance of WT, OsSKL2-OE and OsSKL2-RNAi seedlings on half-strength MS medium under high salinity (120 and 150 mM) and mannitol treatment (200 and 250 mM) at the germination stage. Under control conditions, with no NaCl or mannitol stress, no obvious differences were observed between the WT and transgenic plants. However, after 12 d of respective NaCl and mannitol treatment, the OsSKL2-OE lines exhibited higher relative shoot growth and a greater seminal root number than the WT seedlings. In contrast, the OsSKL2-RNAi lines showed lower relative shoot growth and a lower seminal root number than the WT (Additional file 1: Fig. S5). These results indicate that *OsSKL2* plays a positive regulatory role during osmotic stress in rice at the germination stage.

**Fig. 2:**
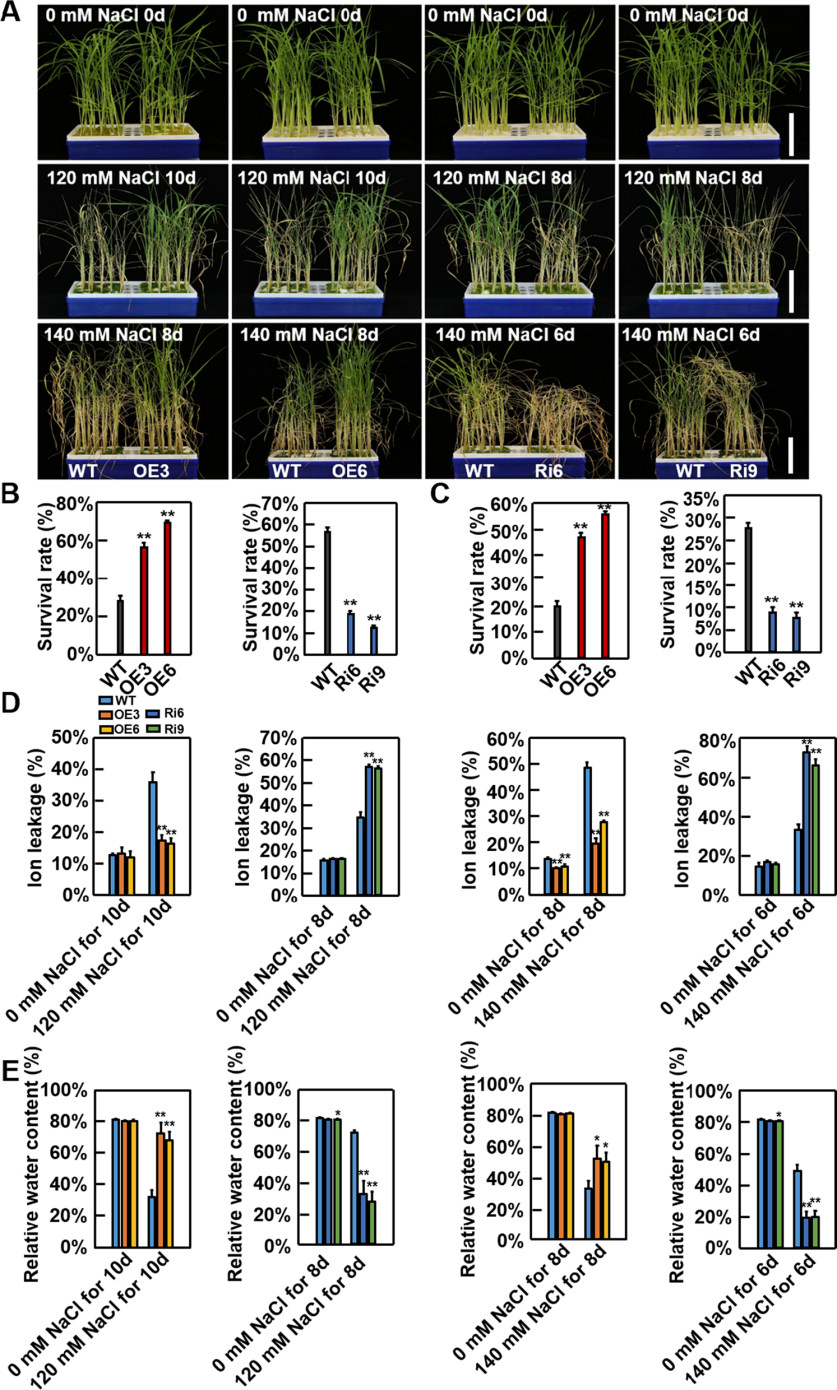
OsSKL2 positively regulates salt tolerance in rice. **A** Phenotypes of wild-type (WT), *OsSKL2*-overexpressing (OE, OE3 and OE6), and RNA interference (RNAi, Ri6 and Ri9) plants before (upper) and after treatment with 120 or 140 mM NaCl (middle and lower images). Two-week-old seedlings were used for salt treatment (bar = 5 cm). Survival rates of WT and *OsSKL2* transgenic plants under **B** 120 and **C** 140 mM NaCl stress. Data represent means ± SD (n = 30). **D** Relative ion leakage and **E** the relative water content of WT and *OsSKL2* transgenic plants under 120 and 140 mM NaCl stress. Data represent means ± SD. Three independent experiments were carried out with similar results. All data were analyzed using one-way analysis of variance (ANOVA) based on the Student’s t-test. *P < 0.05, **P < 0.01

Two-week-old rice seedlings of transgenic and WT plants grown in liquid Hoagland solution were subsequently treated with NaCl to create stress conditions. Under control conditions, no obvious differences were observed between any of the tested lines (Fig. 2A). However, after treatment with 120 mM NaCl for 10 d, the survival rate of the OsSKL2-OE lines was approximately twofold higher than that of the WT plants (Fig. 2B). In contrast, after 8-d treatment with 120 mM NaCl, the survival rate of the OsSKL2-RNAi lines was approximately twofold lower than that of the WT plants (Fig. 2B). A similar trend was also found between the transgenic and WT plants after treatment with 140 mM NaCl (Fig. 2C). In addition, ion leakage and the relative water content (RWC) were also measured in all lines following NaCl treatment. Compared with the WT, salt stress resulted in a significant decrease in relative ion leakage in the OsSKL2-OE plants, but a dramatic increase in the OsSKL2-RNAi plants (Fig. 2D). Meanwhile, the RWC of the OsSKL2-OE plants was significantly higher than that of the WT plants, while that of the OsSKL2-RNAi plants was remarkably lower (Fig. 2E). Next, growth of four-week-old transgenic and WT plants in soil was observed. Compared with the WT, the OsSKL2-OE plants exhibited a higher survival rate and RWC following treatment with 1.5% NaCl, while the OsSKL2-RNAi plants showed a lower survival rate and RWC (Additional file 1: Fig. S6). Taken together, these results demonstrate that *OsSKL2* is crucial for enhancing salt tolerance in rice.

**Fig. 3:**
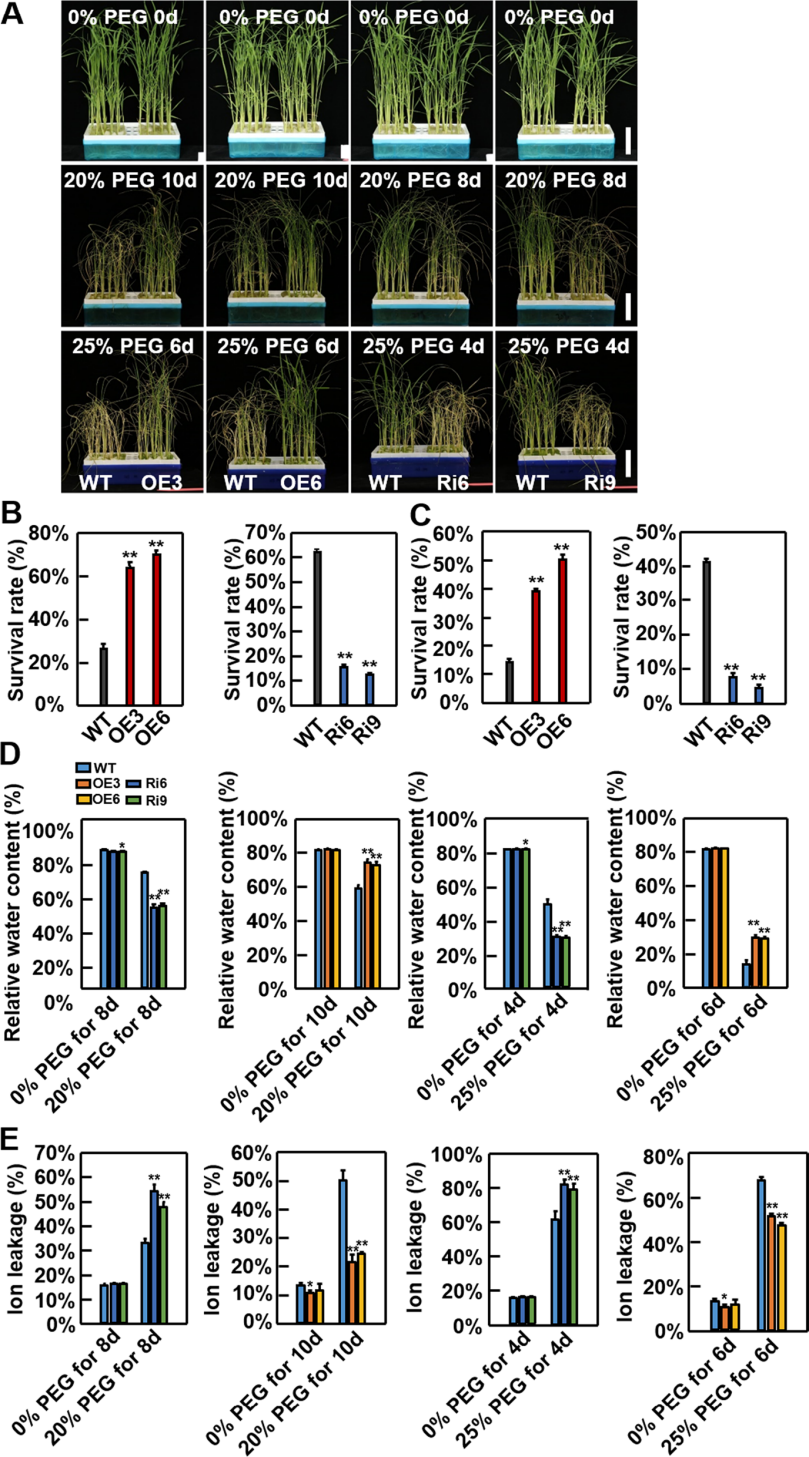
OsSKL2 positively regulates drought tolerance in rice. **A** Phenotypes of wild-type (WT), *OsSKL2*-overexpressing (OE, OE3 and OE6), and RNA interference (RNAi, Ri6 and Ri9) plants before (upper) and after treatment with 20 or25% PEG (middle and lower). Two-week-old seedlings were used for drought treatment (bar = 5 cm). Survival rates of WT and *OsSKL2* transgenic plants under **B** 20 and **C** 25% PEG stress. Data represent means ± SD (n = 30). **D** Relative water contents and **E** relative ion leakage in WT and *OsSKL2* transgenic plants under 20 and 25% PEG stress. Data represent means ± SD. Three independent experiments were carried out with similar results. All data were analyzed using one-way analysis of variance (ANOVA) based on the Student’s t-test. *P < 0.05, **P < 0.01

The function of *OsSKL2* during drought stress was also investigated in liquid Hoagland solution and soil. Under control conditions, no obvious differences were observed between the WT and transgenic lines (Fig. 3A). However, following treatment with 20% PEG for 10 d, the survival rate of the OsSKL2-OE lines was approximately 2.5-fold higher than that of the WT plants (Fig. 3B). In contrast, the survival rate of the OsSKL2-RNAi lines was approximately fourfold lower than that of the WT plants after treatment with 20% PEG for 8 d (Fig. 3B). A similar trend was also found between the transgenic lines and WT plants after treatment with 25% PEG (Fig. 3C). In addition, the RWC of the OsSKL2-OE plants was significantly higher, while that of the OsSKL2-RNAi plants was remarkably lower than the WT (Fig. 3D). Compared with the WT plants, PEG stress also resulted in a significant decrease in relative ion leakage in the OsSKL2-OE plants, but a dramatic increase in the OsSKL2-RNAi plants (Fig. 3E). Four-week-old transgenic and WT plants were subsequently planted in soil. After stopping irrigation then 2-d recovery, the OsSKL2-OE plants exhibited a higher survival rate, while the OsSKL2-RNAi plants presented a lower survival rate compared with the WT (Additional file 1: Fig. S7A & B). In addition, the OsSKL2-OE plants presented a higher RWC and the OsSKL2-RNAi plants a lower RWC compared with the WT (Additional file 1: Fig. S7C). Collectively, these results demonstrate that *OsSKL2* is also important for improving drought tolerance in rice.

### OsSKL2 Improves Oxidative Stress Tolerance in Transgenic Rice

**Fig. 4:**
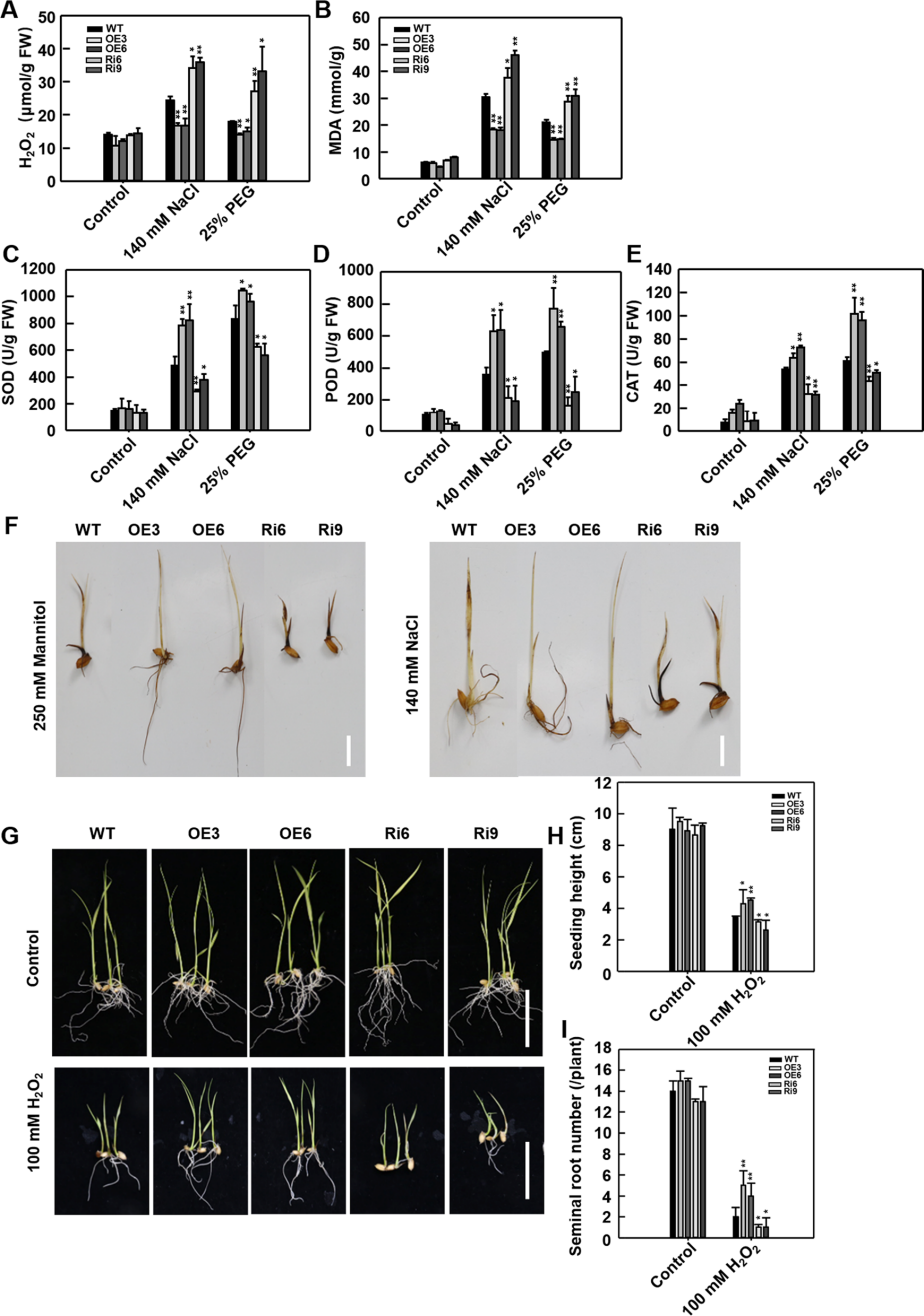
*OsSKL2* promotes tolerance to salt and drought stress by regulating H_2_O_2_ homeostasis in rice. Leaves of four-leaf-stage wild-type (WT) and *OsSKL2* transgenic seedlings were treated with 25% PEG for 5 d or 140 mM NaCl for 7 d then measured for **A** H_2_O_2_, **B** MDA, **C** SOD, **D** POD and **E** CAT enzyme activity. Data represent means ± SD. **F** H_2_O_2_ histochemical analyses of 10-day-old seedlings treated with 250 mM mannitol or 140 mM NaCl then stained with 1% 3,3’-diaminobenzidine tetrachloride (DAB) (bar = 1 cm). **G** Phenotypes of WT and *OsSKL2* transgenic plants before (upper) and after treatment with 100 mM H_2_O_2_ for 10 d (lower) (bar = 5 cm). **H** Seedling height and **I** the seminal root number of WT and *OsSKL2* transgenic plants under 100 mM H_2_O_2_ stress. Data represent means ± SD (n = 30). Three independent experiments were carried out with similar results. All data were analyzed using one-way analysis of variance (ANOVA) based on the Student’s t-test. *P < 0.05, **P < 0.01

Drought and high salinity can induce the production and accumulation of ROS (Foreman et al. 2003). Here, transcriptional levels of *OsSKL2* were rapidly induced following H_2_O_2_ treatment (Fig. 1B), implying that *OsSKL2* plays an important role in oxidative stress. To analyze the role of *OsSKL2* in ROS scavenging, we therefore measured the H_2_O_2_ content of transgenic plants under drought and salt stress conditions. Following treatment, the OsSKL2-OE lines showed a lower H_2_O_2_ level, while the OsSKL2-RNAi lines displayed a higher H_2_O_2_ level than the WT (Fig. 4A). Additionally, several physiological indices related to antioxidant capacity and stress tolerance were also examined. Compared with the WT plants, the OsSKL2-OE lines showed a lower MDA content, and higher SOD, POD and CAT activities, while the OsSKL2-RNAi lines showed a higher MDA content, and lower activities of SOD, POD and CAT under drought and salt conditions (Fig. 4B–E). H_2_O_2_ levels in the WT and transgenic seedlings were subsequently confirmed by 3,3-diaminobenzidine (DAB) staining. Compared with the WT, weaker staining intensity was observed in the leaves and roots of the OsSKL2-OE lines, while the OsSKL2-RNAi lines showed stronger staining (Fig. 4F), suggesting an increase in H_2_O_2_ content. Exogenous H_2_O_2_ was subsequently used to investigate the responses of the *OsSKL2* transgenic lines to oxidative stress. In the absence of H_2_O_2_, no differences in shoot height or root development were observed between the transgenic lines and WT. However, after exposure to 100 mM H_2_O_2_ for 10 d, while the OsSKL2-OE shoots and roots displayed superior growth, development was remarkably suppressed in the OsSKL2-RNAi lines compared with the WT (Fig. 4G–I). Taken together, these results suggest that *OsSKL2* functions to maintain ROS homeostasis in rice.

### OsSKL2 Elevates ABA Sensitivity in Rice

**Fig. 5:**
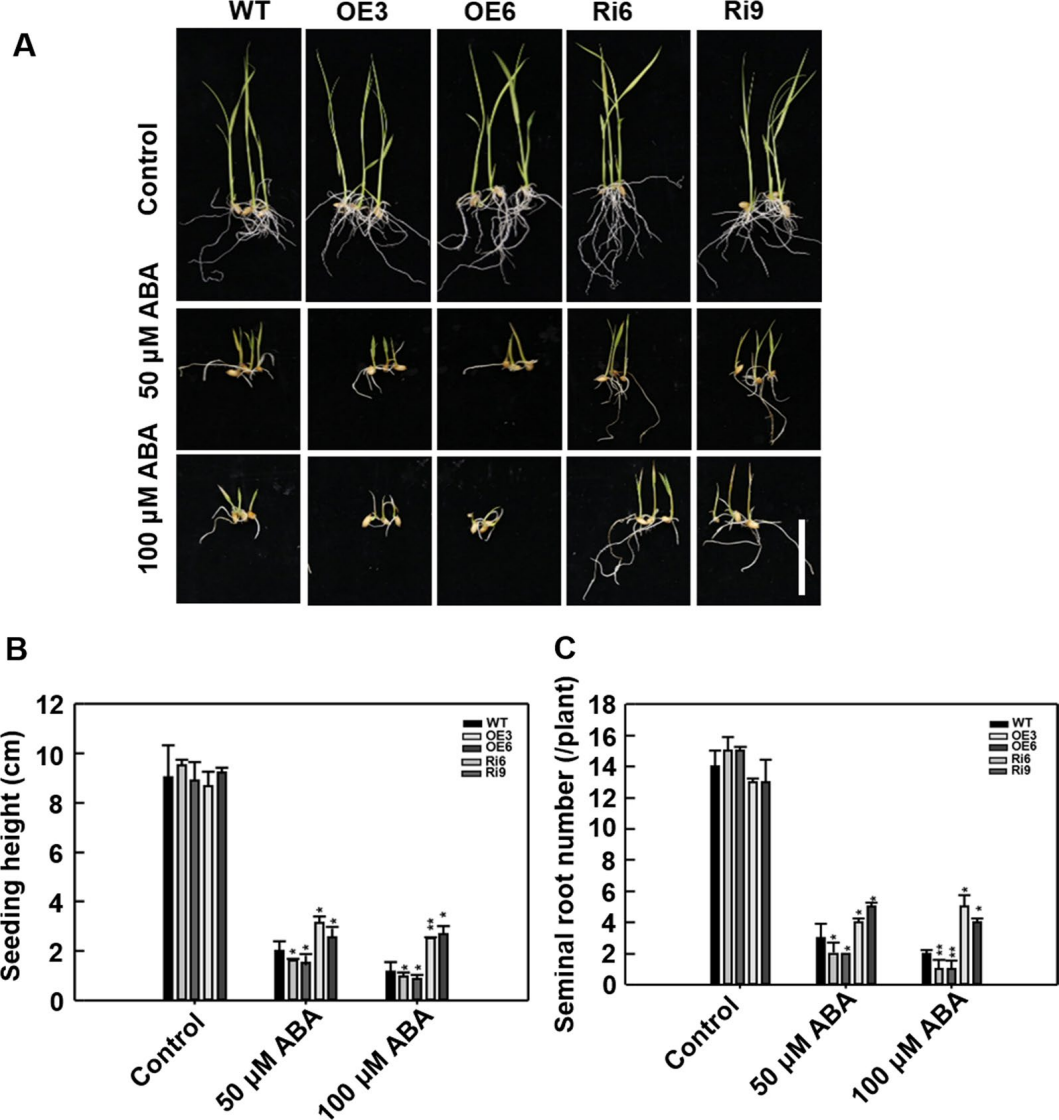
*OsSKL2* increases ABA sensitivity in rice. **A** Phenotypes of WT and OsSKL2 transgenic seedlings grown in 1/2 MS medium with or without 5/10 μM ABA for 10 d (bar = 5 cm). **B** Seedling height and **C** the seminal root number of WT and *OsSKL2* transgenic plants under 5 and 10 μM ABA treatment. Data represent means ± SD (n = 30). Three independent experiments were carried out with similar results. All data were analyzed using one-way analysis of variance (ANOVA) based on the Student’s t-test. *P < 0.05, **P < 0.01

ABA serves as an endogenous messenger during stress responses, with plant drought and salt responses closely related to ABA sensitivity (Yoshida et al. 2014). To determine whether *OsSKL2* is involved in ABA responses, seedling height and root development were examined in the WT and transgenic lines following treatment with exogenous ABA for 10 d. Compared with the WT, significantly reduced shoot and root growth was observed in the OsSKL2-OE lines (Fig. 5A–C), suggesting that growth was arrested by ABA. In contrast, the OsSKL2-RNAi lines exhibited greater shoot growth and a higher root number compared with the WT (Fig. 5A–C), suggesting that less sensitive to ABA. Collectively, these results demonstrate that *OsSKL2* increases sensitivity to ABA, highlighting an important role in the ABA response in rice.

Since ABA induces ROS production (Watkins et al. 2017), we used DAB staining to examine whether exogenous ABA also affected ROS levels in the *OsSKL2* transgenic lines. Under control treatment, there were no obvious differences in ROS accumulation between the WT and *OsSKL2* transgenic lines. However, following treatment with 5 μM ABA for 10 d, strong DAB staining was observed in the leaves of the OsSKL2-OE lines, with weak staining in the OsSKL2-RNAi lines (Additional file 1: Fig. S8). These results suggest that the improved oxidative capability induced by *OsSKL2* is mediated via the ABA regulatory pathway.

### OsSKL2 Interacts With OsASR1

**Fig. 6:**
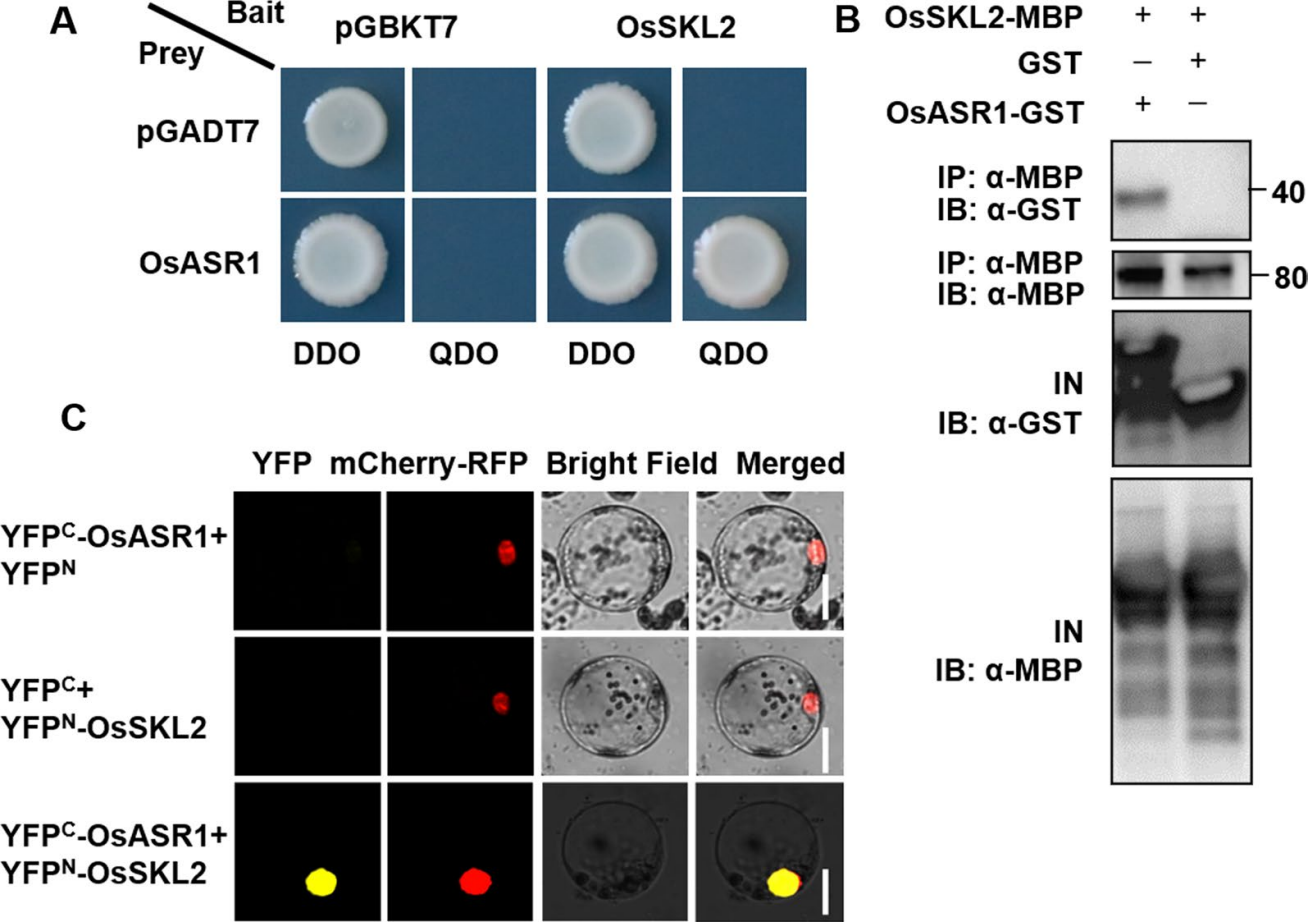
OsSKL2 interacts with OsASR1. **A** OsSKL2 was found to interact with OsASR1 in a yeast two-hybrid system, using BD-OsSKL2 + AD, AD-OsASR1 + BD as negative controls. Growth on selective QDO plates indicated a positive interaction. DDO: yeast SD/-Trp/-Leu selective medium, QDO: yeast SD/-Trp/-Leu/-His/-Ade selective medium. **B** A pull-down assay was then performed to confirm the interaction between OsSKL2 and OsASR1 in vivo. GST and GST-OsASE1 proteins coupled to beads were incubated with MBP-OsSKL2 fusion proteins. Bound OsSKL2 was then detected using mouse anti-MBP antibody. **C** A BIFC assay further revealed that OsSKL2 interacts with OsASR1 in rice leaf protoplasts (bar = 10 μm)

To determine the mechanism by which OsSKL2 modulates drought and salt tolerance, yeast two hybrid screening was performed using full-length OsSKL2 as bait. Several positive clones were obtained and found to correspond to the Abscisic Acid-Stress-Ripening (ASR) protein OsASR1. To verify this interaction, OsSKL2 and OsASR1 were co-transformed into yeast and verified on QDO medium. As expected, yeast containing the OsSKL2 and OsASR1 constructs grew well on QDO medium (Fig. 6A), suggesting interaction between OsSKL2 and OsASR1. To determine the regions of OsSKL2 that interact with the OsASR1 protein, full-length and fragmented OsSKL2 (OsSKL2-N and OsSKL2-C) were examined. As a result, OsSKL2 and OsSKL2-N were found to interact with OsASR1 (Additional file 1: Fig. S9A). Additionally, no interactions between other SK family members (OsSK1, OsSK2, OsSK3 and OsSKL1) and OsASR1 were observed (Additional file 1: Fig. S9B).

To confirm the interaction between OsSKL2 and OsASR1, recombinant proteins were purified in *E. coli* then used in a pull-down assay in vitro. The recombinant OsASR1-GST protein, but not GST alone, was able to pull down the OsSKL2 protein (Fig. 6B), implying a direct interaction between OsSKL2 and OsASR1. Furthermore, bimolecular fluorescence complementation (BiFC) assay was used to further confirm the interaction between OsSKL2 and OsASR1 *in planta*. The BiFC results showed that OsSKL2 specifically interacted with OsASR1, and the YFP signals were detected only following OsSKL2-YFP^N^/OsASR1-YFP^C^ co-transfection of rice protoplasts localized to the nucleus (Fig. 6C). Collectively, these results confirm a direct interaction between OsSKL2 and OsASR1.

### OsASR1 Plays an Important Role in Regulating Salt and Drought Tolerance in Rice

Previous research suggests that *ASR* genes either encode transcription factors or possess chaperone-like activity (Konrad and Bar-Zvi 2008; Arenhart et al. 2014; Li et al. 2017, 2018). To examine this, we characterized the subcellular localization and transcription activation of OsASR1. Both cytosol and nuclear localization of OsASR1 were detected in rice leaf protoplasts (Additional file 1: Fig. S10A). In addition, *OsASR1* possessed no transcription activation activity in yeast (Additional file 1: Fig. S10B). These results suggest that *OsASR1* does not possess the typical features of transcription factors.

Since OsASR1 interacts with OsSKL2, we also examined whether OsASR1 plays a pivotal role in salt and drought stress. To do so, we constructed *OsASR1* OE and RNAi transgenic vectors (Additional file 1: Fig. S11A) then transformed them into WT rice (ZH 11). Several transgenic lines with differing transcription levels were obtained (Additional file 1: Fig. S11B-D) as confirmed by RT-PCR and qRT-PCR. Moreover, since the rice genome contains six *ASR* gene family members, we also examined the transcription levels of other rice *ASR* genes in the OsASR1*-*RNAi lines. As a result, no remarkable reduction in these other *ASR* genes was observed in OsASR1*-*RNAi5 (Ri5) and OsASR1*-*RNAi7 (Ri7) compared with the WT (Additional file 1: Fig. S11E), suggesting that these plants were genuine *OsASR1* knockdown lines. The Ri5 and Ri7 lines, and two OsASR1-OE (OE11 and OE12) lines were therefore selected for the following research.

**Fig. 7:**
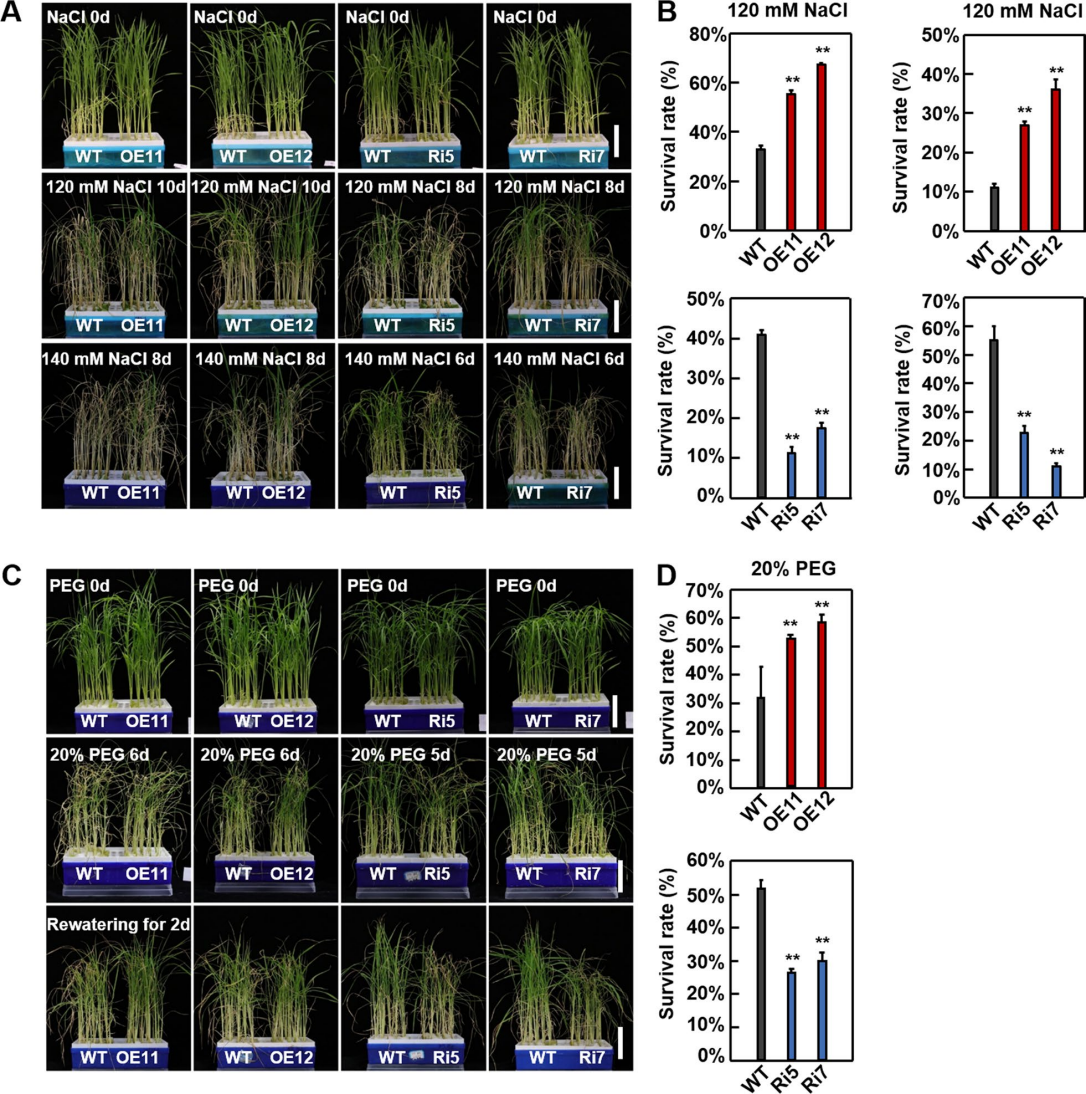
*OsASR1* positively regulates salt and drought tolerance in rice. **A** Phenotypes of wild-type (WT), *OsASR1*-overexpressing (OE, OE11 and OE12), and RNA interference (RNAi, Ri5 and Ri7) plants before (upper) and after treatment with 120 or 140 mM NaCl (middle and lower). Two-week-old seedlings were used for salt treatment (bar = 5 cm). **B** Survival rates of WT and *OsASR1* transgenic plants before and after 120 and 140 mM NaCl stress. Data represent means ± SD (n = 30). **C** Phenotypes of WT and *OsASR1* transgenic plants before (upper) and after treatment with 20% PEG (middle), and after rewatering for 2 d (bottom). Two-week-old seedlings were used for drought treatment (bar = 5 cm). **D** Survival rates of WT and *OsASR1* transgenic plants under drought stress. Data represent means ± SD (n = 30). Three independent experiments were carried out with similar results. All data were analyzed using one-way analysis of variance (ANOVA) based on the Student’s t-test. *P < 0.05, **P < 0.01

To explore the role of *OsASR1* in salt and drought stress, two-week-old seedlings of transgenic and WT plants grown in liquid Hoagland solution were treated with NaCl or PEG. Under control conditions, there were no obvious differences between the transgenic and WT plants (Fig. 7). However, after 10 d treatment with 120 mM NaCl, the OsASR1-OE lines were more robust and had a higher survival rate (58–67%) than the WT plants (32%). Conversely, after 8 d treatment with 120 mM NaCl, the OsASR1-RNAi plants were more wilted and had a lower survival rate (12—17%) than the WT plants (41%) (Fig. 7A, B). Consistent results were also observed between the transgenic and WT plants under 140 mM NaCl treatment (Fig. 7A, B). Meanwhile, under drought conditions, following 6 d treatment with 20% PEG, the WT plants exhibited more severe wilting and a yellowing phenotype than the OsASR1*-*OE lines. Conversely, after 5 d treatment with PEG, the OsASR1-RNAi plants were more severely wilted and had a rolling phenotype compared to the WT (Fig. 7C, D). Moreover, following rewatering, the average survival rates of the two OsASR1*-*OE lines (54 and 57%) were much higher than that of the WT (31%), while those of the two OsASR1*-*RNAi lines (27 and 29%) were much lower than the WT (52%) (Fig. 7C, D). Taken together, these results suggest that *OsASR1* positively regulates salt and drought stress in rice.

### Transient Co-expression of OsSKL2 with OsASR1 Decreased ROS Production

**Fig. 8:**
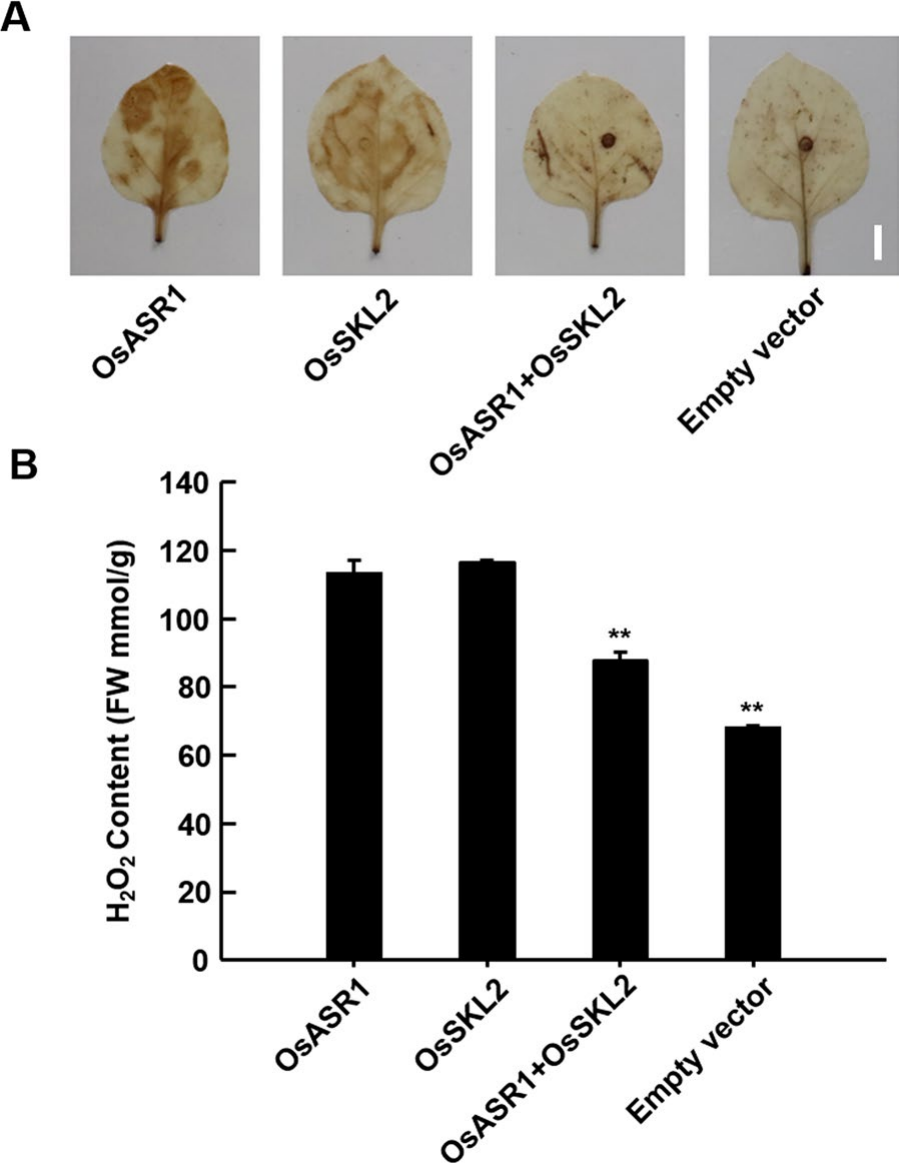
Co-expression of OsSKL2 and OsASR1 decreased ROS production. **A** Transient co-expression of OsSKL2 and OsASR1 in the leaves of *Nicotiana benthamiana.* DAB-stained tobacco leaves were transiently transformed with an empty vector (P35S), OsSKL2 (P35S-OsSKL2), OsASR1 (P35S-OsASR1), and OsSKL2 + OsASR1, respectively (bar = 1 cm). **B** H_2_O_2_ contents of transiently transformed tobacco leaves.Three independent experiments were carried out with similar results. All data were analyzed using one-way analysis of variance (ANOVA) based on the Student’s t-test. **P < 0.01

To explore the possible regulatory mechanism of OsSKL2 and OsASR1 in enhancing tolerance to salt and drought in rice, we carried out transient expression in leaves of *Nicotiana benthamiana*. Compared with leaves expressing an empty vector, strong staining was detected in leaves expressing OsSKL2, OsASR1, and OsSKL2 + OsASR1 (Fig. 8A). However, weaker staining was observed in areas co-expressing OsSKL2 + OsASR1 compared with individual expression (Fig. 8B). These results suggest that transient co-expression of OsSKL2 + OsASR1 caused a reduction in ROS production.

## Discussion

### OsSKL2 Functions as a Novel Positive Regulator of Drought and Salt Tolerance in Rice

The conserved ATP binding domain of most plant SKs consists of a P-loop and Walker B domain, with all sites under purifying selection in these regions (Fucile et al. 2008). In this study, sequence alignment showed that OsSKL2 and AtSKL2 differ dramatically from SK and SKL1 in terms of their P-loop and Walker B domain (Additional file 1: Fig. S1B), suggesting purifying selection of SKL2 families during evolution. In addition, as expected, there were no obvious differences in shikimate content between the WT and *OsSKL2* transgenic plants (Additional file 1: Fig. S4), further suggesting that the two rice SK homologs (*OsSKL1* and *OsSKL2*) evolved from SK gene duplicates and do not possess SK enzyme activity in vitro. These findings indicate that these novel SK-like homologs acquired novel functions during a long period of evolution. In *Arabidopsis*, for example, *SKL1* affects chloroplast biogenesis by regulating auxin pathways (Xu et al. 2018), while *AtSK1* and *AtSK2* were found to show differential expression under biotic and abiotic stress conditions (Fucile et al. 2008). However, direct experimental evidence of the role of *AtSK1* and *AtSK2* in abiotic stress remains largely lacking.

In this study, we identified a novel SK homolog in rice (*OsSKL2*), and revealed a role in responses to drought and salt stress conditions. Since *OsSKL2* expression was strongly induced by mannitol, high salinity, H_2_O_2_ and ABA, positive roles in abiotic stress tolerance were suggested. In line with this, *OsSKL2* overexpressing lines showed tolerance to osmotic stress conditions induced by mannitol and NaCl at the germination stage, while at the post-germination stage, improved survival rates were observed under drought and high salinity treatment induced by PEG and NaCl in both liquid Hoagland solution and soil. In contrast, OsSKL2-RNAi plants showed sensitivity to drought and salt stress (Figs. 2 and 3 & Additional file 1: Fig. S5–7). Importantly, the *OsSKL2* transgenic lines also exhibited no obvious morphological differences under control conditions. Taken together, these results suggest that *OsSKL2* is a novel positive candidate for drought and salt stress tolerance.

### *OsSKL2 Confers to Drought and Salt Stress *via* ROS Scavenging and the ABA Regulatory Pathway*

In plants, drought and high salinity induce the production of ROS; however, excessive accumulation causes cell membrane damage followed by oxidative destruction (Mittler et al. 2011). Accordingly, tolerant species possess a number of ROS detoxifying proteins, such as CAT, POD and SOD, which protect them from oxidative stress (Azevedo-Neto et al. 2006). For example, overexpression of *IbBBX24* and *IbBBX17* in sweet potato improved tolerance to drought and salt stress via scavenging and detoxification of ROS (Zhang et al. 2022), while transgenic *Triticum aestivum* overexpressing *TaPRX-2A* showed increased tolerance to salt as a result of reduced ROS accumulation (Su et al. 2020). In this study, overexpression of *OsSKL2* in rice reduced H_2_O_2_ accumulation and improved the activities of CAT, POD and SOD, thereby enhancing tolerance to drought and salt stress (Fig. 4). In addition, a reduction in MDA content, an indicator of oxidative stress in the membrane lipids, was also observed in OsSKL2-overexpressing plants. In contrast, the OsSKL2-RNAi lines showed reduced tolerance to oxidative stress. Overall, these results suggest that *OsSKL2* is involved in drought and salt tolerance in association with ROS signaling.

Previous research suggests that ABA regulates the production of ROS, altering expression of ROS-scavenging genes (Jiang and Zhang 2002; Milla et al. 2003), while changes in ROS levels were also found to affect ABA signaling and sensitivity (Pastori et al. 2003). FERONIA (*FER*) encodes a receptor-like kinase that regulates ROS accumulation during the ABA response (Yu et al. 2012). In this study, ABA induced ROS accumulation in the OsSKL2-OE lines (Additional file 1: Fig. S8), as well as ABA hypersensitivity (Fig. 5). In contrast, lower ROS accumulation and reduced ABA sensitivity were observed in the OsSKL2-RNAi lines (Additional file 1: Fig. S8 & Fig. 5). These results support, at least partially, a role of ROS as a second messenger in the ABA regulatory pathway (Cho et al. 2009).

### Interaction Between OsSKL2 and OsASR1 Provides New Insight into the Regulatory Mechanisms of Drought and Salt Stress Responses

SKL2 orthologs contain a short amino acid domain similar to the Pfam CS (CHORD and SGT1) domain (Fucile et al. 2008; Additional file 1: Fig. S1C). It was previously reported that the CS domain serves as a binding module during protein–protein interactions (Takahashi et al. 2003; Boter et al. 2007), as supported by our yeast two hybrid assay whereby the CS domain was found to be the key region of the OsSKL2 OsASR1 interaction (Additional file 1: Fig. S9A).

In this study, the SKL2-ASR1 interaction was first suggested in yeast two-hybrid screening then further confirmed by GST pull-down and BIFC assay experiments (Fig. 6). Intriguingly, OsSKL2 was identified as a chloroplast-localized protein (Fig. 1A); however, the BIFC assay showed colocalization of OsSKL2 and OsASR1 in the nucleus of the rice protoplasts (Fig. 6C). These results suggest that OsSKL2 is involved in signaling communication between the chloroplast and nucleus. In line with this, approximately 90% of chloroplast proteins are nuclear encoded and transported across the membrane via specialized proteins such as chaperones (Jarvis and Robinson 2004; Wu et al. 2019). This was also partially demonstrated by the interaction between OsSKL2 and OsASR1 in protein interaction experiments. Moreover, it was further confirmed by the fact that OsASR1 does not possess the typical features of transcription factors, and by the change in OsSKL2 localization in the presence of OsASR1. Overall, these results suggest that OsASR1 serves as a molecular chaperone, assisting OsSKL2 during communication cross-talk between the chloroplast and nucleus.

ROS serve as an important communication signal between the chloroplast and nucleus during abiotic stresses, such as salt and drought (Song et al. 2021). Here, the chloroplast-localized OsSKL2 protein was also found to regulate ROS homeostasis under salt and drought stress (Fig. 4), while interaction between OsSKL2 and OsASR1 was found to positively regulate salt and drought tolerance. These results therefore prompted us to examine whether the SKL2-ASR1 interaction also regulates ROS levels. As expected, transient expression of OsSKL2 + OsASR1 in the leaves of *N. benthamiana* decreased ROS production compared with individual expression (Fig. 8), suggesting that OsSKL2 and OsASR1 enhance tolerance to salt and drought stress by regulating ROS homeostasis. However, whether the interactive mechanism between OsSKL2 and OsASR1, and the mediation of ROS production are dependent signaling mechanisms requires further study.

## Conclusion

In the present study, we identified and characterized a shikimate kinase-like 2 (*SKL2*) gene form rice. Overexpression of *OsSKL2* in rice increased tolerance to salt and drought stresses, whereas transgenic plants of RNAi-induced suppression of *OsSKL2* displayed increased sensitivity to salt and drought treatment. Moreover, OsSKL2 was found to physically interact with OsASR1, which also exhibited positive roles in salt and drought tolerance in transgenic rice. Taken together, the results of this study identified two interacting proteins, OsSKL2 and OsASR1, which act together to positively regulate salt and drought stress in rice, highlighting a potential use in engineering of salt and drought tolerant plants.

## Materials and Methods

### Plant Materials and Stress Treatments

Seeds of WT (*Oryza sativa japonica* cv. Zhonghua11) and transgenic rice plants were placed in a standard growth chamber at 28 ± 2 °C with a 14 h light/10 h dark photoperiod under 70% humidity. Two-week-old WT seedlings were then treated with 100 mM NaCl, 100 mM mannitol, 5 mM H_2_O_2,_ or 100 µM ABA. Root, leaf and stem tissues were then respectively harvested at 0, 1, 3, 9, 12 and 24 h after treatment, immediately frozen in liquid nitrogen and stored at − 80 °C for transcription expression level analysis of *OsSKL2*.

For osmotic stress treatment, T3 rice seeds from transgenic and WT lines were germinated then transplanted in 1/2 MS medium containing 120/150 mM NaCl or 200/250 mM mannitol, respectively. Shoot height and the seminal root number of the transgenic and WT lines were then measured after 12 d treatment. Treatments were replicated in at least three independent biological experiments.

Salt and drought stress at the seedling stage was carried out using two-week-old transgenic and WT lines grown in normal Hoagland solution then treated with 120/140 mM NaCl or 20/25% PEG, respectively. To obtain a more apparent phenotype, the transgenic and WT seedlings were also treated with 120 mM NaCl or 20% PEG for 8 and 10 d, respectively, and with 140 mM NaCl for 6 and 8 d or 25% PEG for 4 and 6 d, respectively. Survival rates, ion leakage and the RWC were then determined from three independent experiments.

For salt and drought treatment in soil, four-week-old transgenic and WT seedlings were grown in nutrient soil (black soil: vermiculite = 1:1). For salt treatment, transgenic and WT seedlings were treated with 1.5% NaCl treatment for 10 and 12 d, respectively. For drought treatment, irrigation was stopped for 10 and 12 d then the seedlings were rewatered for two d. Survival rates and the RWC were then determined from three independent experiments.

For oxidative treatment and the ABA sensitivity assay, seeds of transgenic and WT plants germinated on 1/2 MS medium were transferred to 1/2 MS medium containing 100 mM H_2_O_2_ or 50/100 µM ABA, respectively. After 10 d treatment, seedling height and the seminal root number were measured from three independent experiments.

### Construction of a Plant Vector and Generation of Rice Transgenic Lines

Full-length rice SKL2 (LOC_Os10g42700.1) and ASR1 loci (LOC_Os01g72910.1) were amplified using the following primers: for *OsSKL2*, 5'-GG**GGTACC**ATGTTGGCCTCCACTTGCTTCTCCG-3' and 5'-AACTGCAGTATGTTGGTGGGTGGTGCGTCGGA-3', and for *OsASR1*, 5'-GG**GGTACC**ATGGCTGAGGAGAAGAAG-3' and 5'-AACTGCAGGTATTGGTCGGCGGCGTG-3'. Bold and underlined letters represent KpnI and PstI restriction sites, respectively. The confirmed PCR products were then cloned into the modified pCAMBIA1390 vector in which the original cauliflower mosaic virus (CaMV) 35S promoter was replaced with the maize ubiquitin promoter, and new KpnI and PstI restriction sites were inserted in the MCS region.

Chimeric genes for producing RNA with a hairpin structure (hpRNA) were constructed based on the sequences of rice *SKL2* and *ASR1*, respectively. Specific primers were as follows: for the *OsSKL2* fragment, 5'-ATA**CTCGAG**ACATACCACACGCCCAAGTACG-3' and 5'-ATTAGATCTTCCTCCCCGTCCTTCTTGTG-3', and for the *OsASR1* fragment, 5'-ATA**CTCGAG**GCTGAGGGAATTGGTTACCTTCC-3' and 5'-ATTAGATCTCTGTACGAACATGGCTGCTAAGG-3'. Bold and underlined letters represent XhoI and BglI restriction sites, respectively. The confirmed PCR products were then inserted into the PUC-RNA interference vector in an inverted repeat configuration to generate inverted repeats with an intron space, respectively. The chimeric genes were then cloned into pCAMBIA 1300 under control of the maize ubiquitin promoter to generate *OsSKL2*-RNAi and *OsASR1*-RNAi constructs, respectively. The *OsSKL2* and *OsASR1* overexpressing and RNAi constructs were then introduced into *O. sativa japonica* cv. Zhonghua11 via the *Agrobacterium*-mediated transformation method (Jeon et al. 2000).

### RNA Isolation and qRT-PCR

Total RNA was extracted from rice root, leaf and stem samples using Trizol reagent (TianGen, Beijing, China). First-strand cDNA was then synthesized using SuperScriptTM III reverse transcriptase (Invitrogen, Carlsbad, CA, USA) following the manufacturer’s instructions. Quantitative real-time PCR was performed using the ABI 7500 Real-Time system (Applied Biosystems, USA), with the rice *Actin1* gene as an endogenous control. All experiments were carried out with two biological repeats and three technical trials.

### Analysis of Subcellular Localization

Full-length open reading frames (ORF) of OsSKL2 and OsASR1 without the stop codon were amplified and cloned into the binary pMDC83 vector under control of the CaMV 35S promoter, respectively. The plasmid constructs were then transformed into rice leaves protoplasts and GFP fluorescence signals were observed using a Zeiss LSM 780 confocal laser scanning microscope (Carl Zeiss, Germany) at an argon laser excitation wavelength of 488 nm.

### Physiological and Biochemical Index Assays

The third fully-expanded leaf blade of *OsSKL2* and *OsASR1* overexpressing and RNAi transgenic lines, and WT plants were sampled for analysis of the RWC as follows:

$${\text{RWC}}\left( \% \right) = \left( {{\text{FW}} - {\text{DW}}} \right)/\left( {{\text{TW}} - {\text{DW}}} \right) \times 100$$

where FW represents the fresh weight recorded immediately after sampling, TW represents the turgid weight recorded after soaking in distilled water for 4 h, and DW represents the dry weight recorded after drying at 80 °C for 24 h.

Relative electrolytic leakage (EL) was also measured based on the following method. Briefly, 0.5-g leaf samples were cut into small pieces then placed in tubes containing 50 mL distilled water. An electrical conductivity (EC) meter (Thermo-Scientific™ USA) was then used to determine the EC of the samples. The samples were then autoclaved at 120 °C for 30 min and the EC was re-measured. EL was then calculated as follows:

$${\text{EL }}\left( \% \right) = {\text{C}}1/{\text{C}}2 \times 100.$$

where C1 and C2 represent the first and second EC readings, respectively.

The H_2_O_2_ measurements, ABA content, and enzyme activity assays were determined using leaf samples obtained from four-leaf-stage seedlings treated with 25% PEG for 5 d or 140 mM NaCl for 7 d. H_2_O_2_ was measured using 0.5-g leaf samples homogenized with 5 mL 0.1% TCA then centrifuged at 12,000 g for 15 min. The supernatant was then transferred to a tube containing 0.5 mL 10 mM K_3_PO_4_ and 1 mL 1 M KI, and absorbance at 390 nm was then measured. ABA content was measured using the HPLC–MS/MS method as described previously (Liu et al. 2014; Liang et al. 2019). Briefly, 1-g leaf samples were homogenized in 30 mL of buffer solution (isopropyl alcohol-hydrochloric acid/dichloromethane, 1:2) for 1 h at 4 °C then centrifuged at 12,000 g at 4 °C for 10 min. The precipitate was then dried and dissolved in 100 µL methanol. CAT, POD and SOD activities were measured as described previously (Zhang et al. 2011).

### DAB Staining

For H_2_O_2_ staining, seedlings and transformed tobacco leaves were incubated in 1% DAB (Sigma-Aldrich, MO, USA) in sodium phosphate buffer for 8 h at room temperature then washed three times with washing buffer (ethanol: acetic acid: glycerol = 3: 1: 1) at 95 °C for 15 min. Stained samples were then imaged using a Leica dissecting microscope (DM2500).

### Yeast Two-Hybrid Assays

The MATCHMAKER GAL4 Two-Hybrid System (Clontech, USA) was used for yeast two-hybrid assays according to the manufacturer’s instructions. Briefly, full-length *OsSKL2* was amplified and cloned into the pGBKT7 vector then used to screen the rice cDNA library. Mated yeast cells were then selected on QDO plates and positive clones were used for sequencing.

The interactions between OsASR1 and OsSK1, OsSK2, OsSKL1 and OsSKL2 were then analyzed according to the manufacturer’s protocol. Briefly, the coding regions of *OsSK1*, *OsSK2*, *OsSKL1* and *OsSKL2* were respectively cloned into the pGADT7 vector then the resulting prey constructs were transformed into the yeast strain AH109 together with OsASR1. Yeast cells were then selected on QDO medium for 3–5 days.

### Pull-Down Assay

The full-length coding sequence of *OsASR1* was cloned into the pGEX4T-1 vector to generate GST tag fusion proteins, while the *OsSKL2* coding sequence was cloned into the pMAL™-c2X vector to generate MBP tag fusion proteins. GST, OsASR1-GST, and OsSKL2-MBP were then expressed in *E. coli*. GST and OsASR1-GST respectively immobilized on glutathione sepharose resin were then incubated with OsSKL2-MBP in GST binding buffer (2 mM KH_2_PO_4_, 4.2 mM Na_2_HPO_4_, 10 mM KCl, 140 mM NaCl, 10% bovine serum albumin, pH 7.5) at 4 °C for 2 h. The resulting GST beads were then washed three times with buffer (2 mM KH_2_PO_4_, 4.2 mM Na_2_HPO_4_, 10 mM KCl, 140 mM NaCl) and analyzed by immunoblotting with anti-MBP antibody or anti-GST antibody (Abmart, Shanghai, China).

### BIFC Assay

The coding sequences of OsSKL2 and OsASR1 were cloned separately into p2YN-35S and p2YC-35S vectors containing YFP fragments to form OsSKL2-YFP^N^ and OsASR1-YFP^C^ fusion proteins, respectively. The constructs were then co-transformed into rice protoplasts (Chen et al. 2006) and visualized fluorescently using a confocal laser scanning microscope (Carl Zeiss780).

### Statistical Analyses

Statistical analyses were carried out using one-way analysis of variance (ANOVA) using Student’s t-test, followed by Bonferroni’s post-hoc test. All results are expressed as the mean ± standard deviation (SD) of at least three replicates.

## Supplementary Information

**Additional file 1: Fig. S1.** Phylogenetic relationships and sequence analysis of SK genes. **A** Phylogenetic tree constructed using MEGA7.0 based on the N-J method. Bootstrap values (above 50%) from 1000 replicates are indicated at each node. **B** Sequence alignment of conserved motifs of SK and SK-like homologs. **C** CS domain analysis in the AtSKL2 and OsSKL2 proteins. **Fig. S2.** Tissue expression profiles of OsSKL2 in rice root, stem and leaf. **Fig. S3.** Plasmid construction and expression of OsSKL2 in the transgenic rice lines. **A** Schematic diagram of the RNAi construct used for the development of OsSKL2 transgenic rice. **B** Expression levels of OsSKL2 in the wild-type (WT), OsSKL2 overexpressing (OE3 and OE6), and OsSKL2 RNAi (RI6 and RI9) transgenic lines as determined by qRT-PCR. OsActin1 was used as an RNA loading standard for comparison of OsSKL2 expression levels. C Expression levels of OsSKL1 and OsSKL2 in the OsSKL2-RNAi lines. **Fig. S4.** Analysis of the shikimic acid contents of wild-type (WT) and OsSKL2 transgenic plants. **Fig. S5.** OsSKL2 enhanced tolerance to osmotic stress at the germination stage. **A** Phenotypes of wild-type (WT) and OsSKL2 transgenic seeds germinated on 1/2 MS medium with or without 120/150 mM NaCl or 200/250 mM mannitol for 12 d, respectively (bar = 5 cm). **B** Seedling height and **C** the Seminal root number of WT and OsSKL2 transgenic plants before and after osmotic treatment. Data represent means ± SD (n = 36). Three independent experiments were carried out with similar results. All data were analyzed using one-way analysis of variance (ANOVA) based on the Student’s t-test. *P < 0.05, **P < 0.01. **Fig. S6.** OsSKL2 enhanced salt tolerance in rice grown in soil. **A** Phenotypes of wild-type (WT) and OsSKL2 transgenic seedlings before and after treatment with 1.5% NaCl. Four-week-old seedlings were used for NaCl treatment (bar = 10 cm). **B** Survival rates and **C** relative water contents of WT and OsSKL2 transgenic plants before and after treatment with 1.5% NaCl (n = 30). **Fig. S7.** OsSKL2 enhanced drought tolerance in rice grown in soil. **A** Phenotypes of wild-type (WT) and OsSKL2 transgenic seedlings before and after drought treatment, and after re-watering Four-week-old seedlings were used for drought treatment. (bar = 10 cm). **B** Survival rates and **C** relative water contents of WT and OsSKL2 transgenic plants before and after drought treatment (n = 30). **Fig. S8.** Comparison of ROS accumulation following treatment with ABA. Leaves of wild-type (WT) and OsSKL2 transgenic lines stained with DAB to show ROS accumulation following exposure to 5 μM ABA for 10 d (bar = 1 cm). **Fig. S9.** Interactions between OsSKL2, other SK homologs and OsASR1 based on a yeast two-hybrid assay. **A** Interactions between various fragments of OsSKL2 and OsASR1. **B** Interactions between OsASR1 and other rice SK homologs. **Fig. S10.** Subcellular localization and transcription activation assay of OsASR1. **A** Analysis of the subcellular localization of OsASR1 using a rice protoplast transient transformation system (bar = 10 μm). **B** Transactivation activity analysis of full-length OsASR1 in yeast. **Fig. S11.** Plasmid construction and expression levels of OsASR1 in the transgenic rice lines. **A** Schematic diagram of the RNAi construct used for OsASR1 transgenic rice. **B**–**D** Expression levels of OsASR1 in the wild-type (WT) and OsASR1 transgenic lines as determined by RT-PCR and qRT-PCR. **E** Expression levels of other rice ASR members in the OsASR1-RNAi lines. OsActin1 was used as an RNA loading standard for comparison of OsASR1 expression levels.

## Data Availability

All data supporting the findings of this study are available from the corresponding author on reasonable request.
