# Supplementary figures and images for: Regulation of Drought and Salt Tolerance by OsSKL2 and OsASR1 in Rice
Source: Rice (N Y). 2022 Aug 29;15:46. doi: 10.1186/s12284-022-00592-2 (PMC9424430; doi:10.1186/s12284-022-00592-2)

**Fig. S1**

**
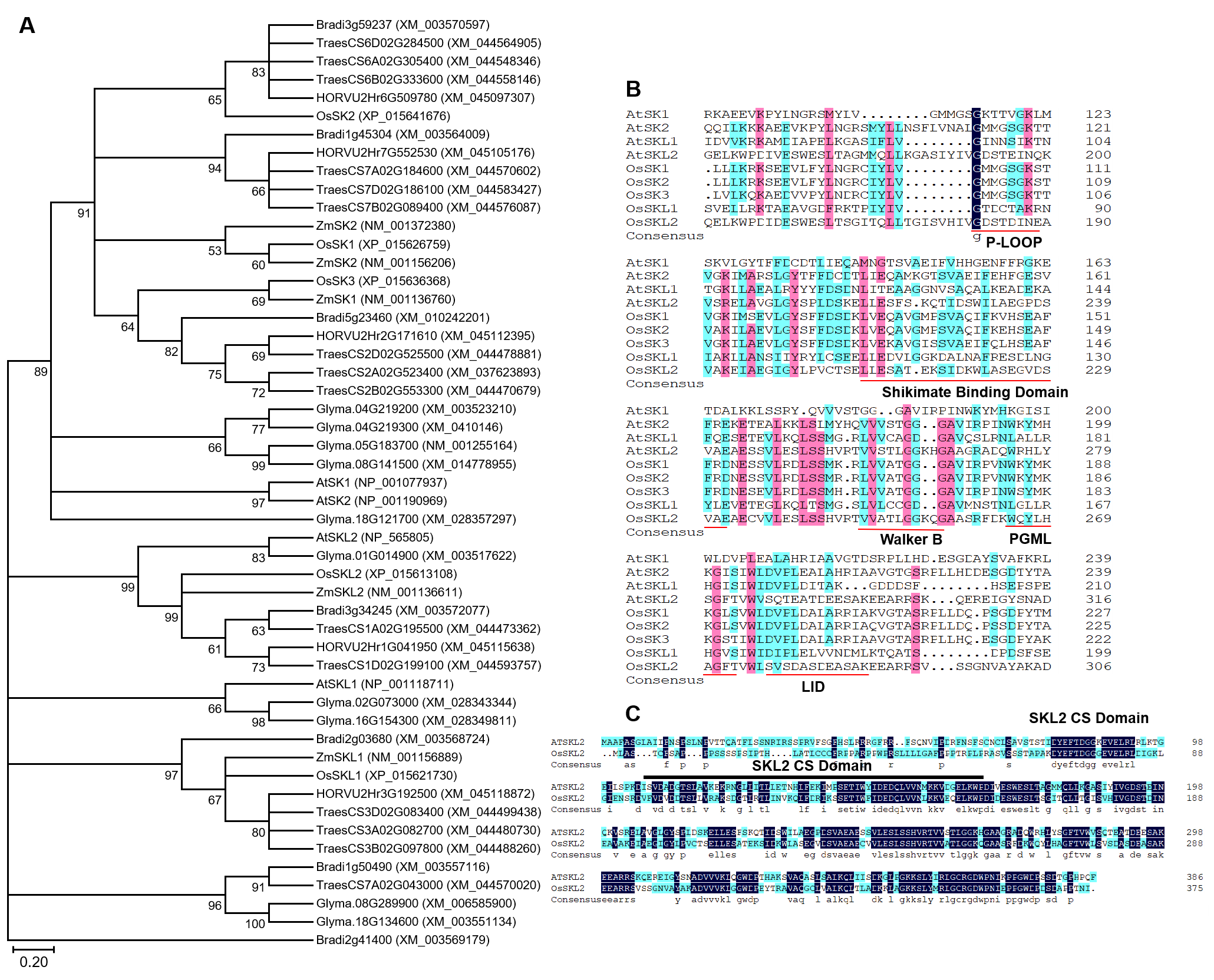
**

**Fig. S2**

**
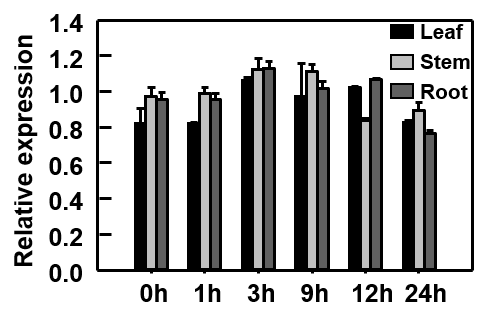
**

**Fig. S3**

**
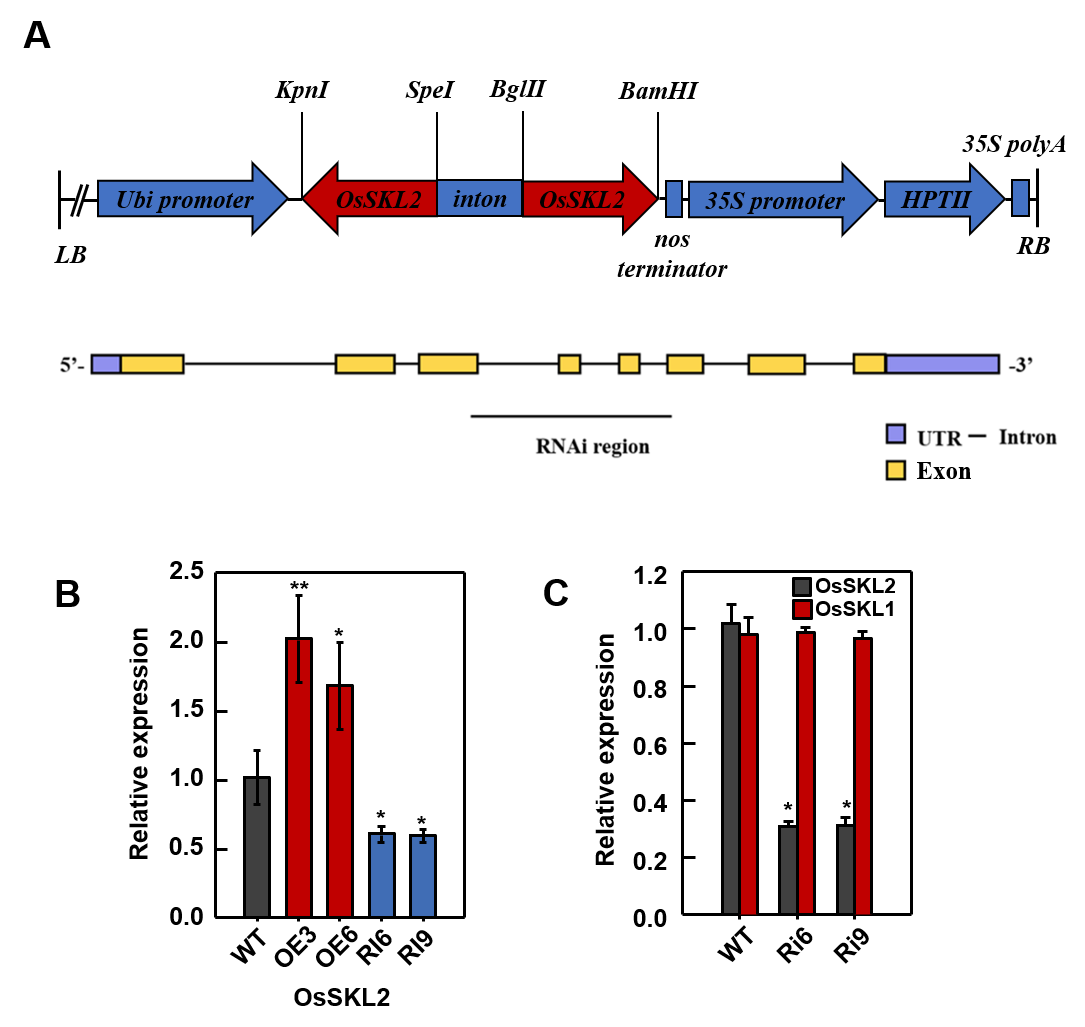
**

**Fig. S4**

**
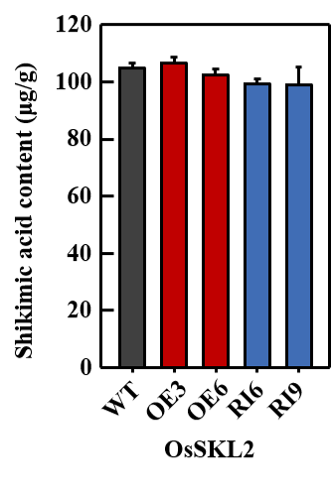
**

**Fig. S5**

**
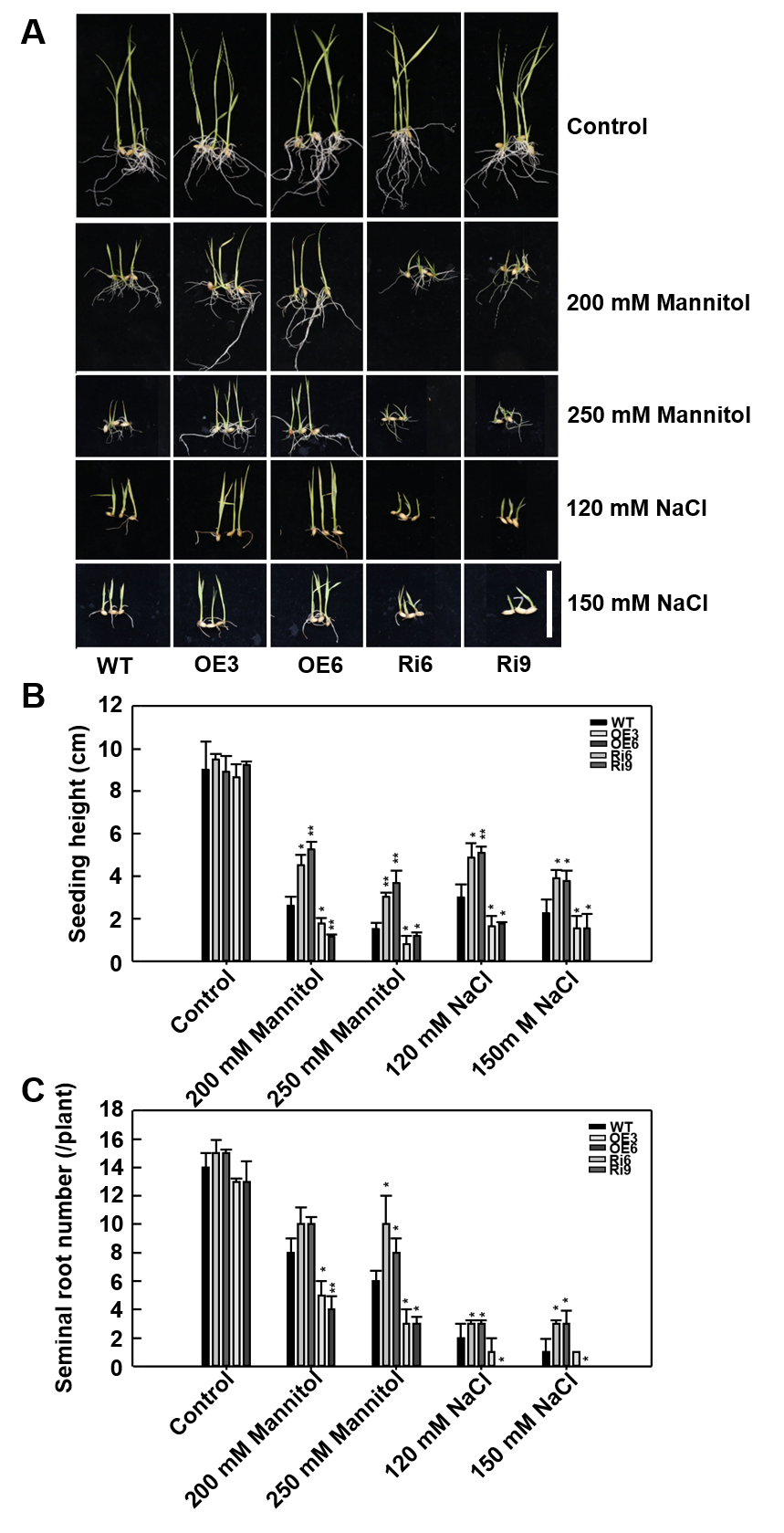
**

**
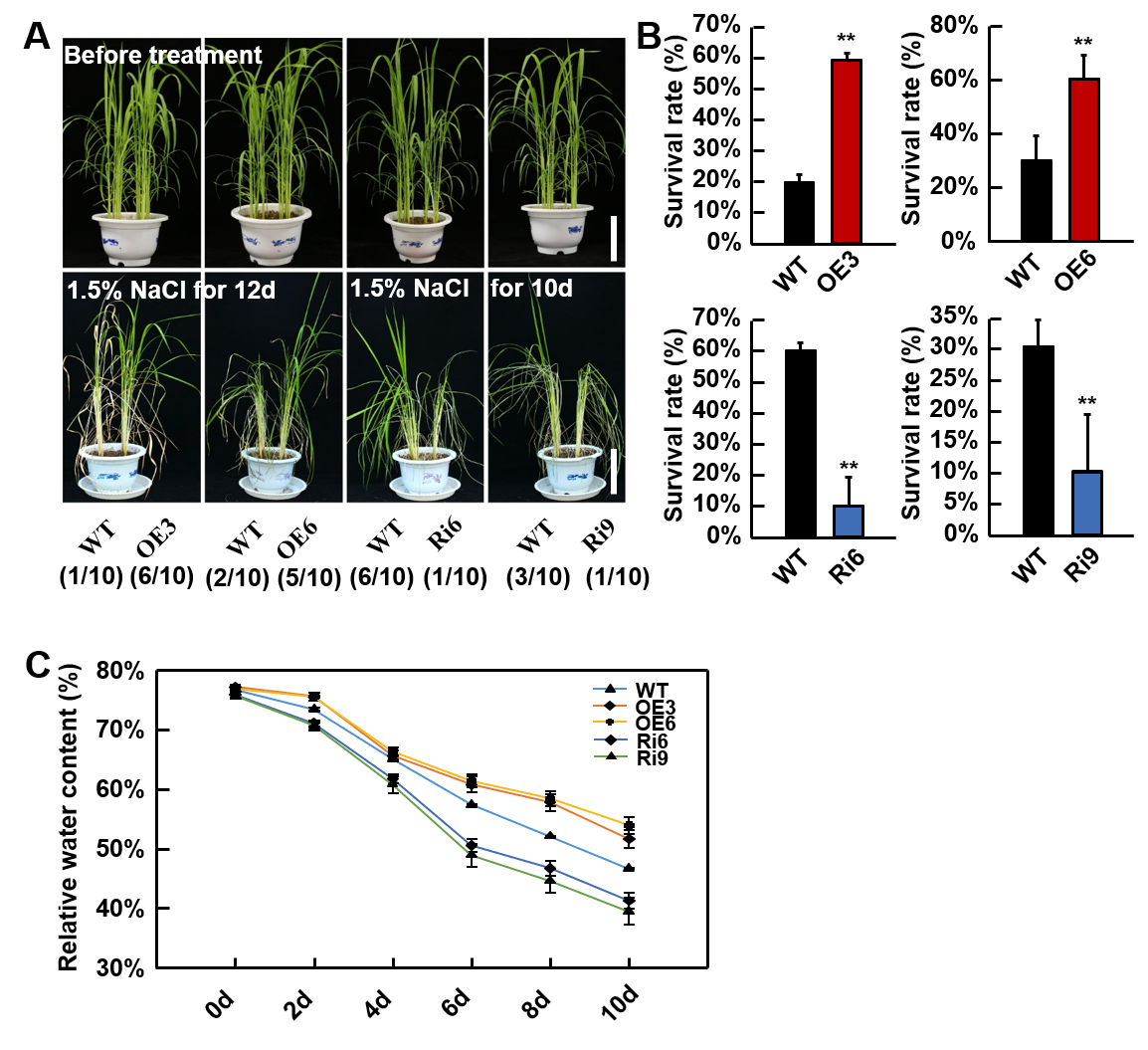
**

**Fig. S6**

**
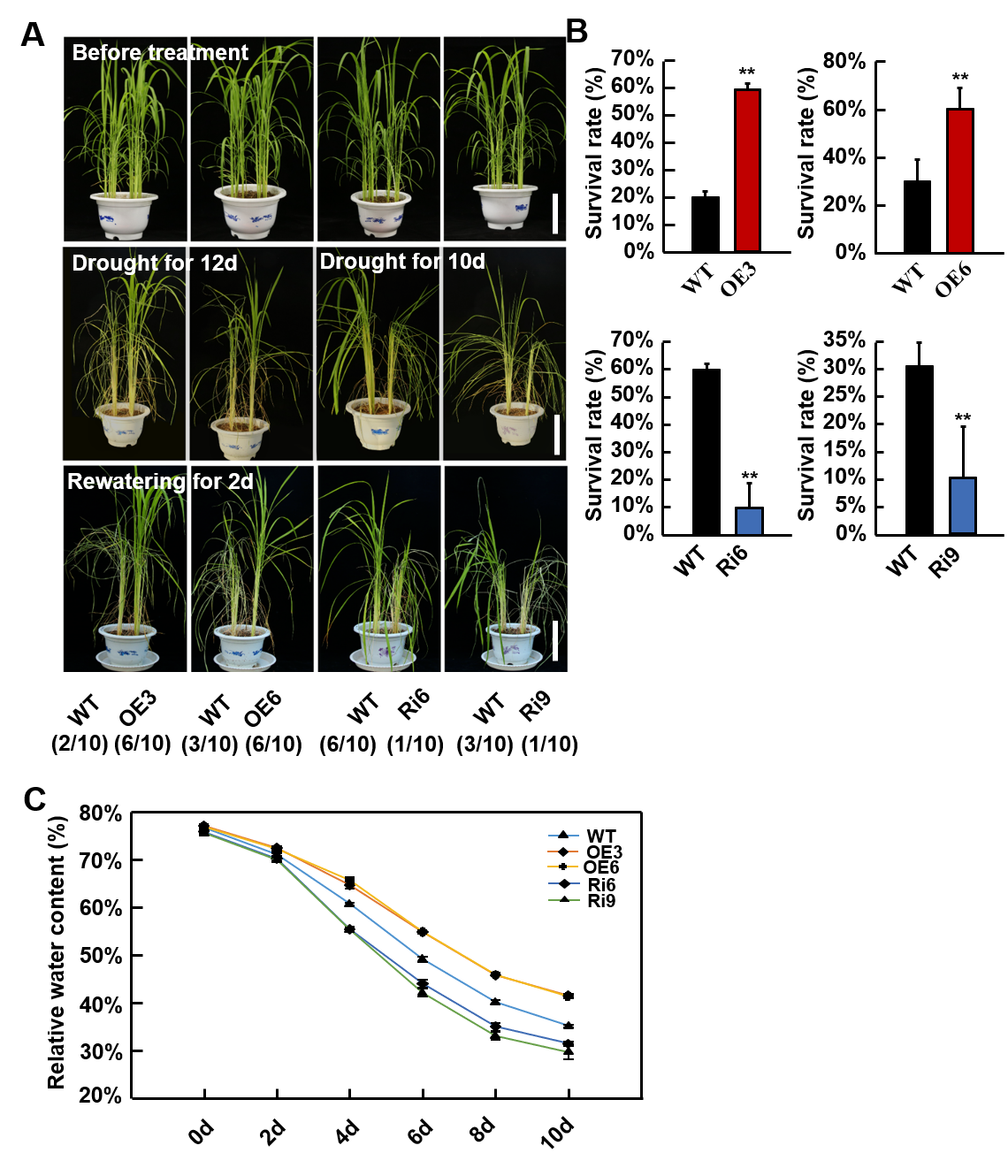
**

**Fig. S7**

**
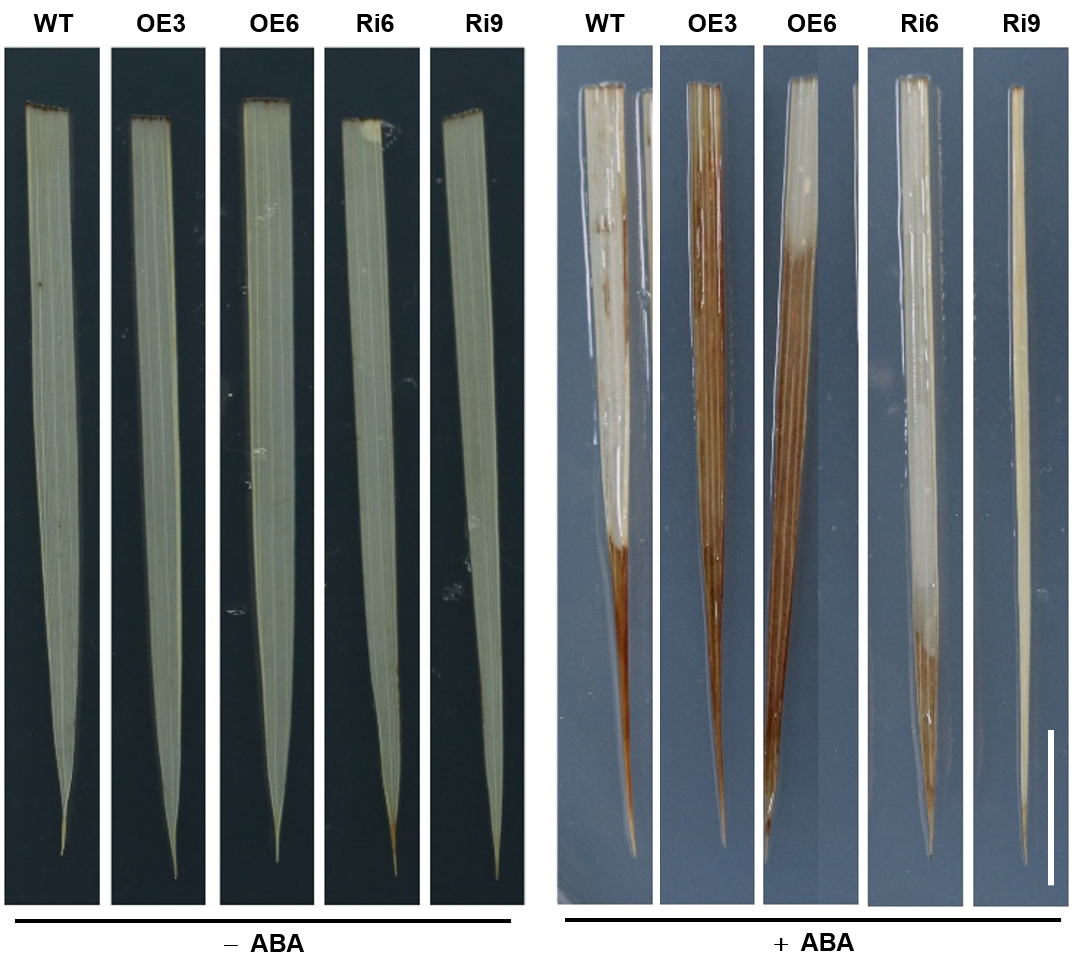
**

**Fig. S8**

**Fig. S9
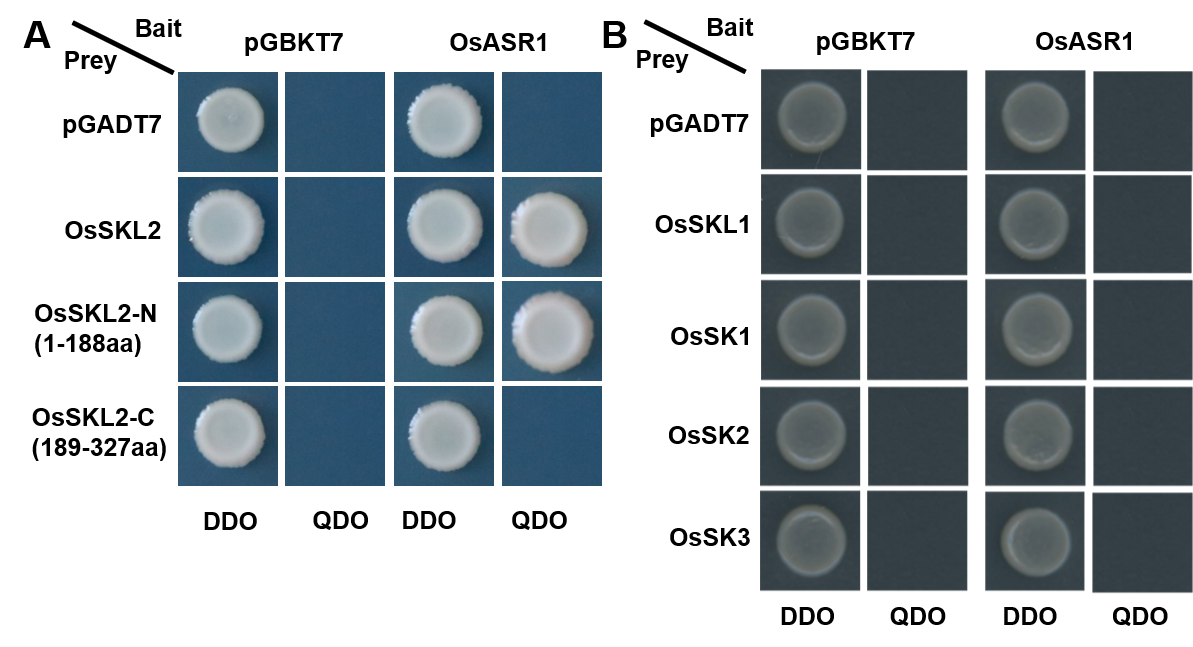
**

**Fig. S10**

**
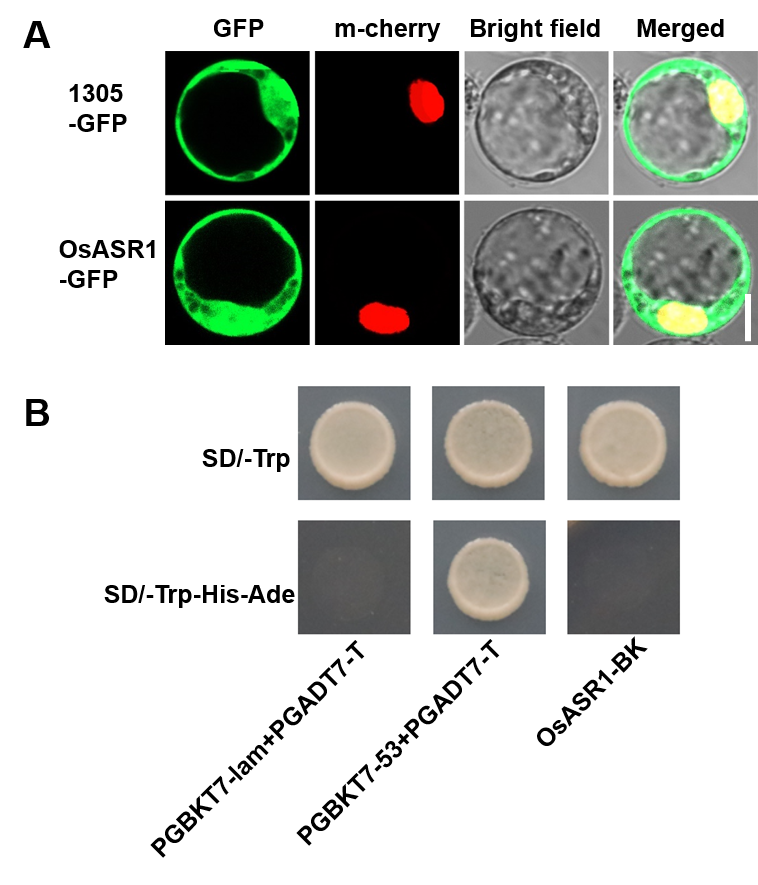
**

**Fig. S11
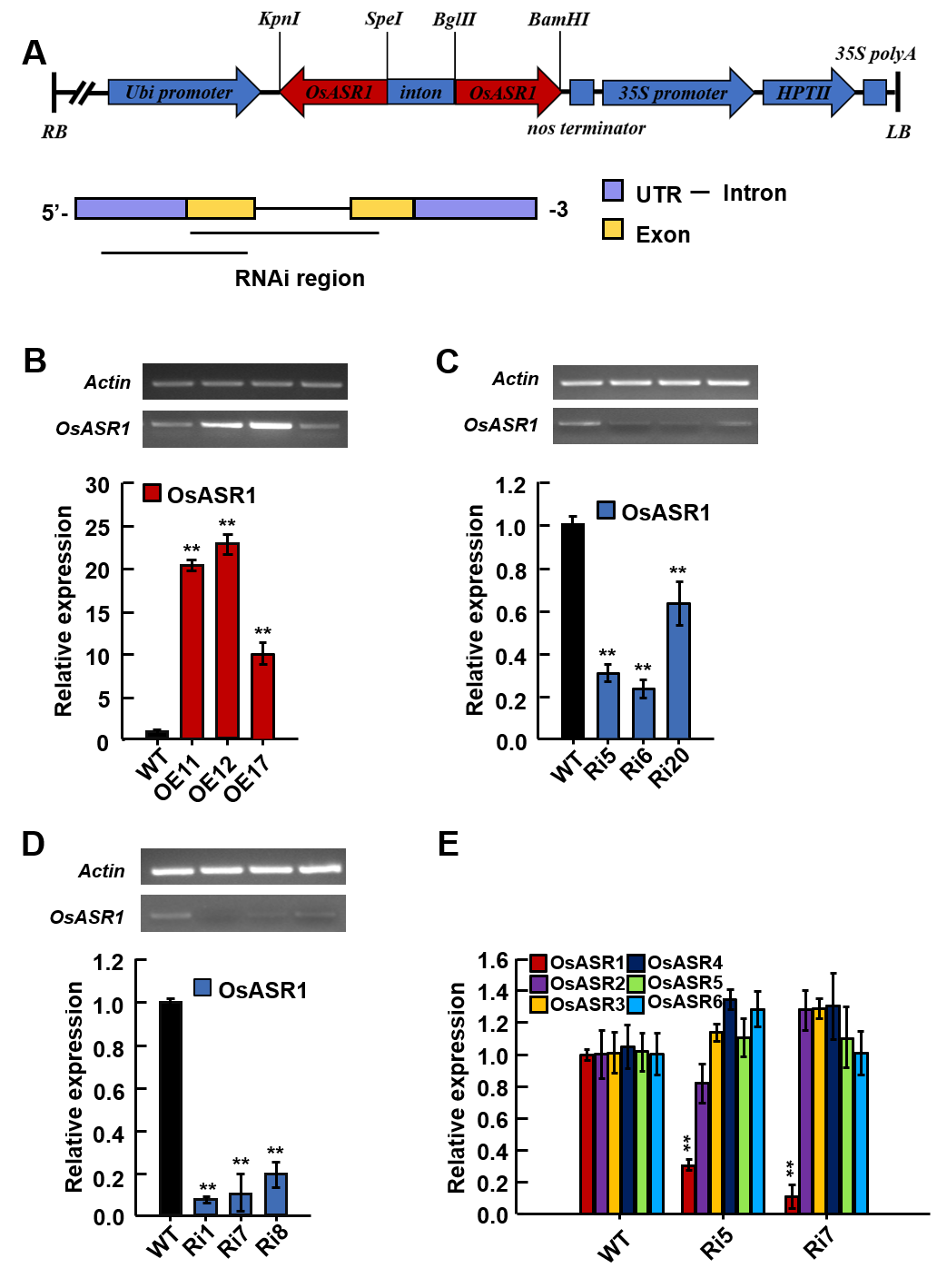
**

Supplement: Supplementary file 1 — Additional file 1: Fig. S1. Phylogenetic relationships and sequence analysis of SK genes. A Phylogenetic tree constructed using MEGA7.0 based on the N-J method. Bootstrap values (above 50%) from 1000 replicates are indicated at each node. B Sequence alignment of conserved motifs of SK and SK-like homologs. C CS domain analysis in the AtSKL2 and OsSKL2 proteins. Fig. S2. Tissue expression profiles of OsSKL2 in rice root, stem and leaf. Fig. S3. Plasmid construction and expression of OsSKL2 in the transgenic rice lines. A Schematic diagram of the RNAi construct used for the development of OsSKL2 transgenic rice. B Expression levels of OsSKL2 in the wild-type (WT), OsSKL2 overexpressing (OE3 and OE6), and OsSKL2 RNAi (RI6 and RI9) transgenic lines as determined by qRT-PCR. OsActin1 was used as an RNA loading standard for comparison of OsSKL2 expression levels. C Expression levels of OsSKL1 and OsSKL2 in the OsSKL2-RNAi lines. Fig. S4. Analysis of the shikimic acid contents of wild-type (WT) and OsSKL2 transgenic plants. Fig. S5. OsSKL2 enhanced tolerance to osmotic stress at the germination stage. A Phenotypes of wild-type (WT) and OsSKL2 transgenic seeds germinated on 1/2 MS medium with or without 120/150 mM NaCl or 200/250 mM mannitol for 12 d, respectively (bar = 5 cm). B Seedling height and C the Seminal root number of WT and OsSKL2 transgenic plants before and after osmotic treatment. Data represent means ± SD (n = 36). Three independent experiments were carried out with similar results. All data were analyzed using one-way analysis of variance (ANOVA) based on the Student’s t-test. *P < 0.05, **P < 0.01. Fig. S6. OsSKL2 enhanced salt tolerance in rice grown in soil. A Phenotypes of wild-type (WT) and OsSKL2 transgenic seedlings before and after treatment with 1.5% NaCl. Four-week-old seedlings were used for NaCl treatment (bar = 10 cm). B Survival rates and C relative water contents of WT and OsSKL2 transgenic plants before and after treatment with 1.5% NaCl (n = [file 12284_2022_592_MOESM1_ESM.docx]
